# Histidine-Covalent Stapled Alpha-Helical Peptides Targeting hMcl-1

**DOI:** 10.1021/acs.jmedchem.4c00277

**Published:** 2024-05-02

**Authors:** Giulia Alboreggia, Parima Udompholkul, Carlo Baggio, Kendall Muzzarelli, Zahra Assar, Maurizio Pellecchia

**Affiliations:** † Division of Biomedical Sciences, School of Medicine, University of California Riverside, 900 University Avenue, Riverside, California 92521, United States; ‡ Cayman Chemical Co., 1180 E. Ellsworth road, Ann Arbor, Michigan 48108, United States

## Abstract

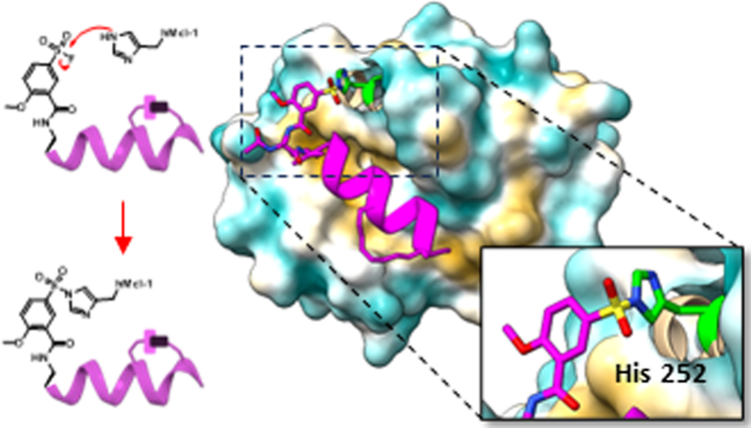

Several novel and effective cysteine targeting (Cys) covalent drugs are in clinical use. However, the target area containing a druggable Cys residue is limited. Therefore, methods for creating covalent drugs that target different residues are being looked for; examples of such ligands include those that target the residues lysine (Lys) and tyrosine (Tyr). Though the histidine (His) side chain is more frequently found in protein binding locations and has higher desirable nucleophilicity, surprisingly limited research has been done to specifically target this residue, and there are not many examples of His-targeting ligands that have been rationally designed. In the current work, we created novel stapled peptides that are intended to target hMcl-1 His 252 covalently. We describe the in vitro (biochemical, NMR, and X-ray) and cellular design and characterization of such agents. Our findings further suggest that the use of electrophiles to specifically target His residues is warranted.

## Introduction

The design of novel pharmacological tools targeting protein–protein interactions (PPIs) has long been regarded as a challenging task. To this end, when PPIs are mediated by an alpha-helix, helix stabilizing strategies have been proposed, some of which have led to agents that have entered clinical trials.^1−7^ We recently reported on novel ligand discovery strategies to target PPIs that included mixture-based screening strategies, such as the HTS by NMR and related approaches.^8,9^ More recently, we also focused on strategies to derive Lys-covalent PPI antagonists^10,11^ using aryl-fluorosulfates or aryl-sulfonyl fluorides. In a recent application, we derived a novel BH3 peptide targeting covalently Lys 234 of the antiapoptotic Bcl-2 protein hMcl-1.^12^

hMcl-1 is often overexpressed in cancer, where it confers resistance to chemotherapy or radiation. Small molecule inhibitors have emerged for hMcl-1,^13−16^ and research is still ongoing in this target space.^9,16−21^ In addition to small molecule inhibitors, as anticipated above, one approach that holds great therapeutic potential in targeting PPIs is the design of stapled alpha-helical peptides.^1,7^ These could provide alternative strategies for advancing novel therapeutic agents or for the design of pharmacological tools for basic research and target validation studies. However, in targeting PPIs with stapled alpha-helices, potency, selectivity, solubility, and cell penetration are all issues that need to be addressed. Given our recent studies in designing covalent PPI antagonists targeting lysine side chains and the intrinsic pharmacodynamics and pharmacokinetics advantages of covalent agents over reversible compounds,^22−25^ this current study aimed at deriving for the first time histidine-covalent BH3-based hMcl-1 targeting agents. His residues are particularly attractive yet underexplored for covalent drug design given that they, unlike Lys, are usually unprotonated at physiological pH, which should facilitate a nucleophilic addition at the imidazole ring.^26^ Moreover, His side chains are considerably more rigid than Lys, allowing, in principle, more effective juxtaposition of electrophiles such as aryl-sulfonyl fluorides or aryl-fluorosulfates^26,27^ and are often found in protein-binding sites.^28^ Despite these favorable theoretical properties, there are only a few reports on rationally designed His-covalent agents.^27,29,30^ We will describe the strategies used to design and derive those agents and the associated characterizations in vitro and in cellular assays.

## Results

### Design and Synthesis of Covalent Stapled alpha-Helical BH3 Peptides Targeting hMcl-1(172–323)

**Figure 1:**
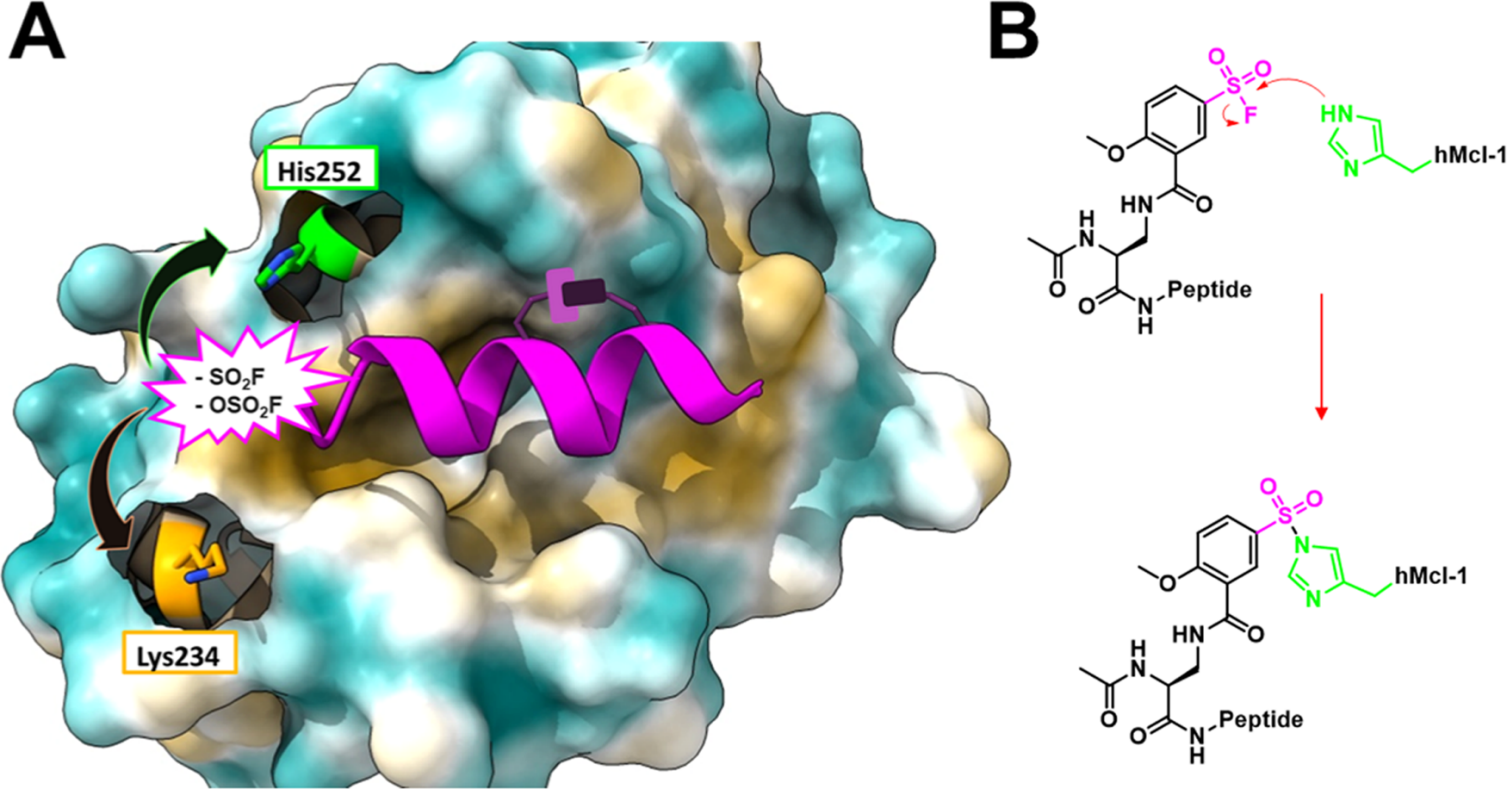
Schematic representation of the proposed strategy to derive stapled alpha-helical-based covalent hMcl-1(172–323) targeting agents. (A) Schematic representation of hMcl-1(172–323) and of the position of the covalent stapled peptide in the binding pocket. The side chains of residues Lys 234 and His 252 are highlighted. The X-ray structure of our previously reported Lys covalent peptide in complex with hMcl-1(172–323) (PDB ID 6VBX) was used.^12^ The surface and ribbon representation was prepared with the software ChimeraX.^32^ (B) Schematic representation of the interaction between hMcl-1(172–323) His 252 residue (green) and the electrophile (2-methoxy 5-sulfonyl fluoride) on the side chain of a 3 amino propionic acid present in the peptide’s structure.

**Figure 2:**
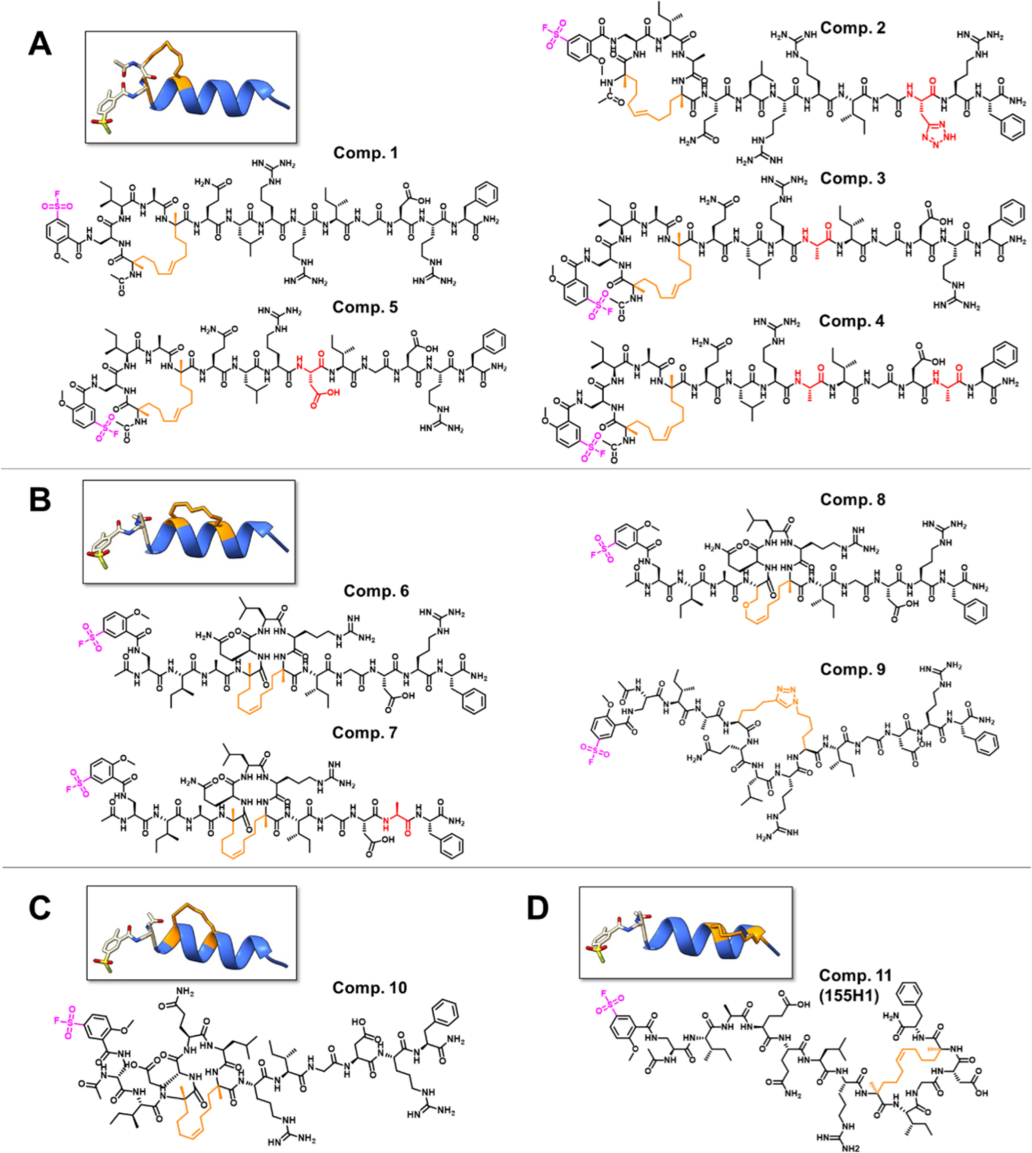
Design of stapled hMcl-1(172–323) targeting peptides. Four different strategies were attempted to place the staple in a region of the peptide that is solvent-exposed, not to alter the binding properties of the resulting constrained alpha-helical peptide to hMcl-1(172–323) (see also Table 1). (A) Hydrocarbon staple was generated by placing (*S*)-2-(4-pentenyl)Ala at positions 1 and 5; (B) hydrocarbon staples were generated by placing either (*S*)-2-(4-pentenyl)Ala or the azido-Lys and l-bis-homopropargylglycine in positions 5 and 9; (C) Hydrocarbon staple was generated by placing (*S*)-2-(4-pentenyl)Ala in positions 4 and 8; (D) hydrocarbon staple was generated by placing (*S*)-2-(4-pentenyl)Ala in positions 9 and 13. Our recently determined X-ray structure of hMcl-1(172–323) in the complex with **138E12** (Table 1) was used to generate the models (PDB ID 6VBX) and identify the possible positions of the staples.^12^

Recently, we have examined the X-ray structure of hMcl-1(172–323) in complex with a BIM-derived BH3 peptide (PDB ID 2NL9) and identified hMcl-1(172–323) Lys 234 as a possible residue for covalent modification. The introduction of aryl-sulfonyl fluorides was accomplished after various rounds of structure–activity relationship studies that led to compound **16** (138E12) (Table 1).^12^ Structural characterization of this agent by various biophysical means confirmed the covalent adduct formation between the peptide and Lys 234, as, among others, revealed by the X-ray structure of the complex with hMcl-1(172–323) (PDB ID 6VBX).^12^ However, in a position opposite Lys 234, the side chain of His 252 is, in principle, an even more attractive residue for covalent modifications, given the nucleophilic properties of the imidazole (Figure 1). Hence, rigidification of the alpha-helical peptide via stapling strategies could juxtapose an electrophile to either Lys 234 or His 252 (Figure 1). Based on previous experience with hMcl-1(172–323) and with an artificial model system, we used to assess covalent His targeting,^27^ we introduced a 2-methoxy 5-sulfonyl fluoride^31^ on the side chain of a 3 amino propionic acid to introduce the possible electrophile for His/Lys covalent adduct formation (Figure 1), in addition to constraining the alpha-helix with chemical staples at various positions (Figure 2). We chose the 2-methoxy 5-sulfonyl fluoride electrophile as our recent studies indicated that it possesses a proper balance between reactivity and stability, and when incorporated in ligands targeting the BIR3 domain of XIAP resulted in Lys covalent compounds that were stable and cell permeable.^31^

**Table 1:** List of Synthesized Stapled Peptides and Sequence of Linear BIM(146–166) and 138E12 Peptides^a^

name	structure	molecular weight (Da)
**BIM**	IWIAQELRRIGDEFNAYYARR-CONH_2_	2639
**16 (138E12)**	Ac-Dap(2MeO,5FSB)IAEQLRRIGDRF-CONH_2_	1815
**1**	Ac-**X**-Dap(2MeO,5FSB)IA**X**QLRRIGDRF-CONH_2_	1937
**2**	Ac-**X**-Dap(2MeO,5FSB)IA**X**QLRRIG(3-(2H-Tetrazol-5-yl)Ala)RF-CONH_2_	1961
**3**	Ac-**X**-Dap(2MeO,5FSB)IA**X**QLRAIGDRF-CONH_2_	1851
**4**	Ac-**X**-Dap(2MeO,5FSB)IA**X**QLRAIGDAF-CONH_2_	1766
**5**	Ac-**X**-Dap(2MeO,5FSB)IA**X**QLRDIGDRF-CONH_2_	1895
**6**	Ac-Dap(2MeO,5FSB)IA**X**QLR**X**IGDRF-CONH_2_	1780
**7**	Ac-Dap(2MeO,5FSB)IA**X**QLR**X**IGDAF-CONH_2_	1695
**8**	Ac-Dap(2MeO,5FSB)IA**B**QLR**X**IGDRF-CONH_2_	1768
**9**	Ac-Dap(2MeO,5FSB)IA**Z**QLR**O**IGDRF-CONH_2_	1807
**10**	Ac-Dap(2MeO,5FSB)I**X**EQL**X**RIGDRF-CONH_2_	1838
**11 (155H1)**	Ac-Dap(2MeO,5FSB)IAEQLR**X**IGD**X**F-CONH_2_	1753

a **X** = (*S*)-2-(4-pentenyl)Ala; **O** = Lys(N_3_); **Z** = l-bis-homopropargylglycine; **B** = allyl-l-serine; 2MeO,5FSB = 2-methoxy, 5-sulfonyl fluoride (see also Figure 2).

Hence, several such stapled peptides were designed, as reported in Figure 2 and Table 1. These agents were synthesized by using conventional solid-phase peptide synthesis protocols, followed by an on-resin ring-closing metathesis reaction or click reaction (Supporting Figures S1 and S2). Using this strategy, it was possible to cross-link the side chains of appropriately modified amino acids that should constrain the given peptide in an alpha-helical conformation without altering their binding properties for hMcl-1(172–323).

The IC_50_ values obtained using a DELFIA assay,^33^ which was used to measure the ability of each agent to displace a reference biotinylated-BIM BH3 peptide, are presented in Table 2, together with the net charge value of each agent, and the percentage of alpha-helix conformation as derived from circular dichroism (CD) measurements for each agent. Structure–activity relationship studies followed typical trends of passing from linear to stapled peptides, with the position of the staple influencing the affinity for the target. This may be due to either the conformational constraints imposed by the staple, steric hindrances between the staple and the target or elimination of intermolecular interactions replacing amino acids with those composing with the staple. Hence, obvious staple positions that are predicted based on the X-ray structure of 138E12 in complex with Mcl-1 to be incompatible with its binding mode were not considered. Similarly, we also introduced slight variations in the side chains that are predicted to be solvent-exposed in the complex, to varying net charge of the resulting peptides (Table 2). Hence, we noted that stapling the linear peptide at the tested position resulted in peptides that largely retained the binding affinity for Mcl-1, with the exception of compounds **2** and **10** that were significantly less potent than the linear molecule. In compound **2**, this is not entirely surprising given that we attempted (unsuccessfully) to replace the peptide aspartic acid residue (involved in a critical salt bridge in the complex with Mcl-1) with the possible bioisostere tetrazolyl-Ala (Figure 2). Similarly, the arginine in position 7 of the peptide was also identified as important for the interactions with Mcl-1 in our previous SAR studies;^12^ hence, not surprisingly, compound **10**, which introduces the hydrocarbon staple in the place of this residue, is less active than the linear peptides. DELFIA data indicated that all of the remaining agents, in particular compounds **4**, **6**, **7**, **8**, and **11** (**155H1**), were as potent or more potent than the linear peptide in targeting hMcl-1(172–323).

**Table 2:** In vitro Characterization of Stapled and Linear Peptides^a^.

name	IC_50_ (nM) hMcl-1(172–323)	net charge	% alpha-helix
**BIM**	3.0 ± 0.0	+1	ND
**138E12**	18 ± 4	+1	48.3^b^
**1**	24 ± 3	+2	100
**2**	633 ± 30	+3	100
**3**	35 ± 3	+1	100
**4**	17 ± 2	0	66.1
**5**	30 ± 2	0	100
**6**	14 ± 1	+1	100
**7**	12 ± 2	0	73.5
**8**	6.6 ± 0.3	+1	93.5
**9**	21 ± 3	+1	100
**10**	174 ± 1	0	100
**11 (155H1)**	18 ± 3	–1	100^c^

a IC_50_ values obtained by a DELFIA displacement assay (*n* = 2), net charge, and percentage of alpha-helix calculated using BeStSel^34^ are reported.

b CD spectra were measured both in water and in 15 mM phosphate buffer pH 7.5.

c CD spectra were measured in 15 mM phosphate buffer pH 7.5. ND = Not determined.

**Figure 3:**
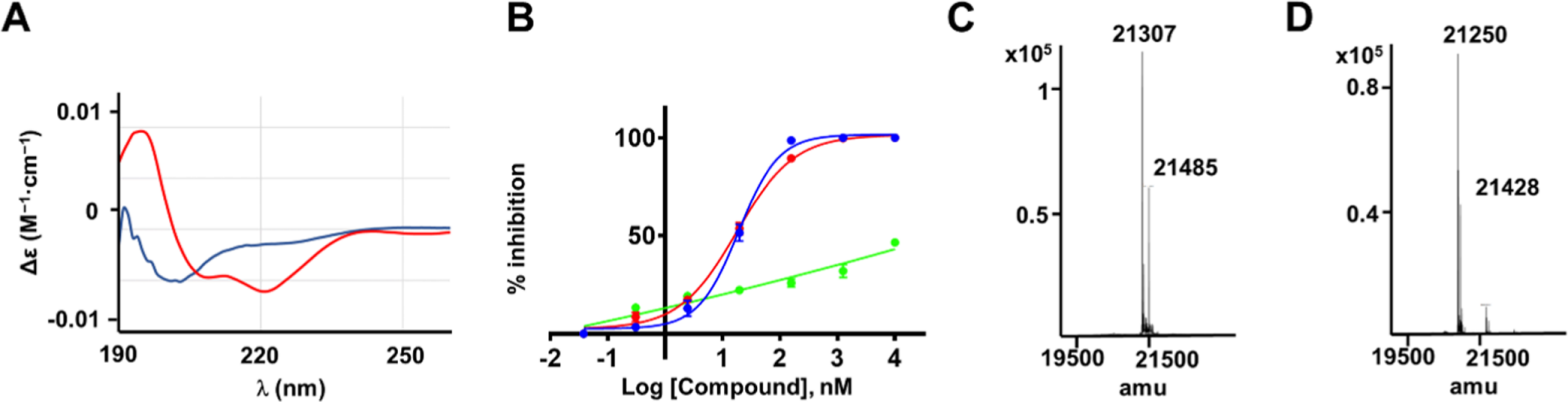
Characterization of stapled hMcl-1(172–323) targeting covalent peptides. (A) Representative of CD curves obtained for the linear compound **138E12** (blue) and for stapled peptide **155H1** (red). (B) Example of dose–response DELFIA displacement assay curves comparing the ability of linear **138E12** (blue), with stapled peptide **155H1**, to displace the binding of a biotinylated-BH3 peptide to hMcl-1(172–323) (red) or hBfl-1(1–149) (green) (see Experimental Section). (C) LC-MS analysis performed to confirm the formation of the covalent complex. Ten μM hMcl-1(172–323) were incubated with 100 μM **155H1** overnight (16 h) at room temperature in a buffer (50 mM phosphate pH 7.5, 150 mM NaCl, 1 mM DTT). The mass of hMcl-1(172–323) is 19 572 Da, which is increased by 1735 Da (1753–20 Da (HF = leaving group)) in the presence of **155H1**, corresponding to the anticipated mass of the covalent adduct of 21 307 Da. 21 485 Da (21 307 Da + 178) corresponds to the mass after phosphogluconoylation of the target (MW hMcl-1(172–323) = 19 750 Da) (mass data for hMcl-1(172–323) are reported in Figure S17). (D) As in part (C) but with the Lys234Ala mutant of hMcl-1(172–323). The mass of hMcl-1(172–323) mutant is 19 515 Da, which is increased by 1735 Da (1753–20 Da (HF = leaving group)) in the presence of **155H1**, corresponding to the anticipated mass of the covalent adduct of 21 250 Da. 21 428 Da (21 250 Da + 178) corresponds to the mass after phosphogluconoylation of the target (MW hMcl-1 K234A(172–323) = 19 693 Da) (mass data for K234A hMcl-1(172–323) data are reported in Figure S18).

Furthermore, to verify that the peptides preferentially assumed a stabilized alpha-helical conformation in the stapled version compared to the linear peptide, we performed CD measurements. Typical curves for the linear peptide (**138E12**, blue) and a stapled peptide (**155H1**, red) are reported in Figure 3, while CD data for all other peptides are reported as the Supporting Information (Supporting Figures S3–S14). Using these measurements and the software BeStSel (https://bestsel.elte.hu),^34^ we could calculate the percentage of alpha-helical content for each peptide (Table 2). Most stapled peptides, perhaps except compound **4** and compound **7**, assumed nearly complete alpha-helical conformation in aqueous buffer (Table 2 and Supporting Figures S3–S14). Similar to what we had previously observed with compound **138E12**,^12^ our agents were significantly weaker in targeting the closely related Bcl-2 family protein hBfl-1(1–149) (Figure 3).^12^ This is in part because the starting peptide was designed to be considerably weaker for Bfl-1(1–149) to begin with (see also example Supporting Figure S15)^12^ and also because hBfl-1(1–149) does not present equivalent Lys or His residues that could covalently interact with our agents.^12^ Compounds **4** and **7** presented limited aqueous solubility, which also affected the quality of the CD curves obtained.

**Figure 4:**
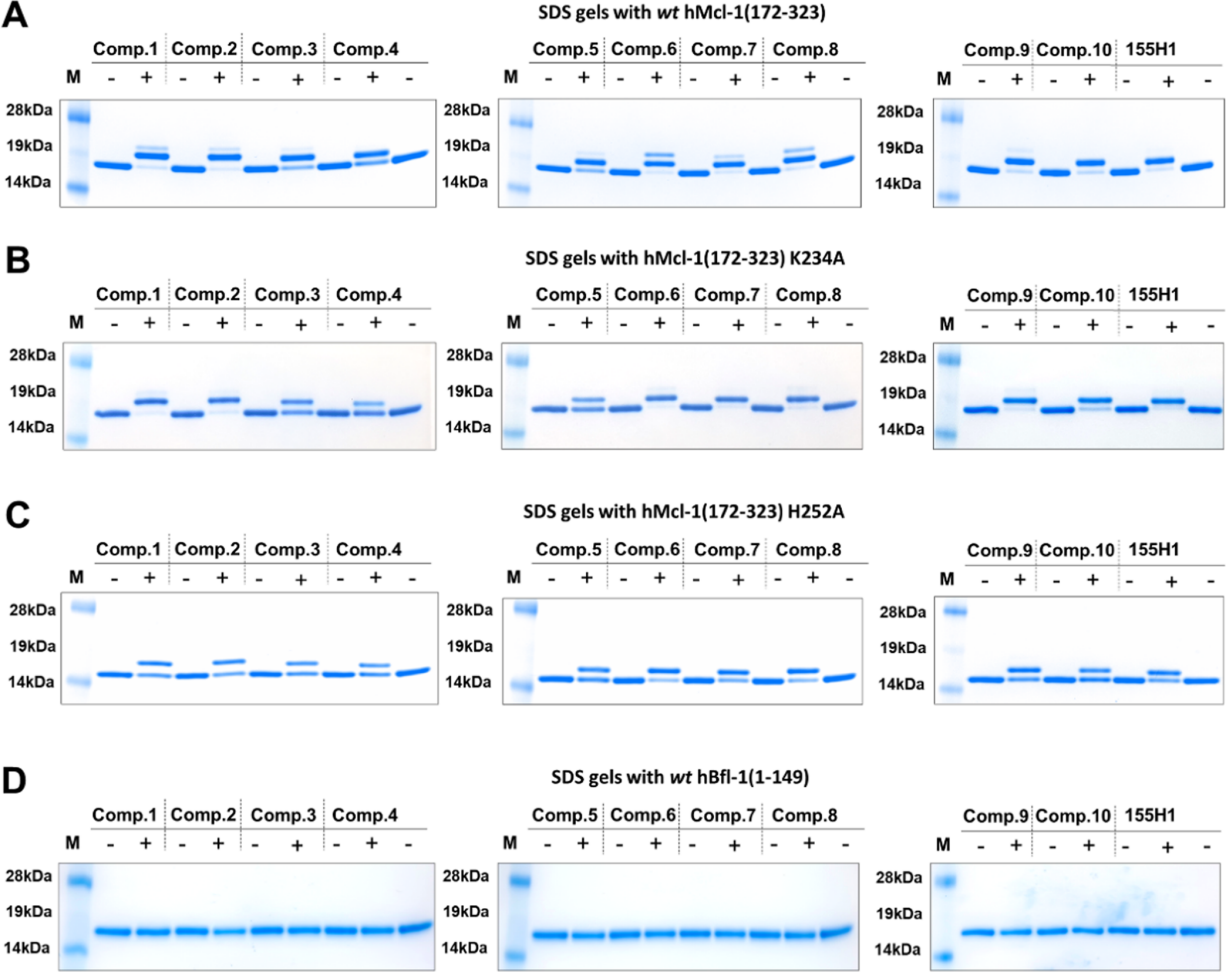
Stapled peptides target hMcl-1(172–323) covalently and selectively. (A) SDS gel electrophoresis of wt HisTag hMcl-1(172–323) collected in the absence or presence of each stapled peptide. The samples for the gels were obtained by incubating 10 μM wt HisTag hMcl-1(172–323) with 100 μM of the indicated agent overnight (16 h) at room temperature in a buffer (50 mM phosphate, pH 7.5, 150 mM NaCl, 1 mM DTT). (B) As in part (A) but with a Lys234Ala mutant of 6xHis-hMcl-1(172–323). (C) As in part (A) but with a His252Ala hMcl-1(172–323) (no histidine tag present). (D) As in part (A) but with wt 6xHis-hBfl-1(1–149).

To verify that each agent was targeting hMcl-1(172–323) covalently, targeting either Lys 234 or His 252, we used mass spectrometry measurements and SDS gel electrophoresis. Hence, the MW of the adduct was verified via mass spectrometry with both wt hMcl-1(171–323) and a Lys234Ala hMcl-1(171–323) mutant (Figure 3C,D), suggesting that our agents bound covalently to hMcl-1(172–323). The data, however, suggest that His 252 is the likely target for our agents, given that the covalent adduct formation was detected with both wt and the Lys234Ala hMcl-1(172–323) mutant (Figure 3D). Moreover, purified recombinant hMcl-1(172–323) was exposed to each compound (at a ligand/protein ratio of 1:10) for 16 h prior to characterization of the complex via SDS gel electrophoresis. Given the difference in MW of the target in the free versus bound state, a gel shift was expected when a stable (SDS- and heat-resistant) covalent adduct was formed (Figure 4A). Subsequently, we conducted the same experiment with a Lys234Ala hMcl-1(172–323) mutant (Figure 4B). Most agents presented a gel shift indicative of covalent adduct formation with both *wt* and Lys234Ala mutant hMcl-1(172–323), again suggesting that the electrophile may be covalently interacting with His 252 (Figure 4A,B). However, as anticipated earlier (Figure 1), the agents can also interact covalently with Lys 234 when His 252 was mutated to Ala (Figure 4C). Conversely, when the same experiment was conducted with hBfl-1, no band shift was observed (Figure 4D), consistent with the low affinity of the agents for hBfl-1(1–149) and the selectivity of the electrophile, considering that hBfl-1(1–149) contains 7 His residues and 10 Lys residues. A time-course for the reaction between **155H1** and Mcl-1(172–323) using SDS-Gel electrophoresis and MS analyses was also conducted, indicating that the reaction half-life of about 6 h, under the experimental conditions: ligand/protein ratio 1:10 at room temperature in buffer (50 mM phosphate pH 7.5, 150 mM NaCl, 1 mM DTT) (Supporting Figure S16).

### Nuclear Magnetic Resonance (NMR)-Based Ligand-Binding Studies

**Figure 5:**
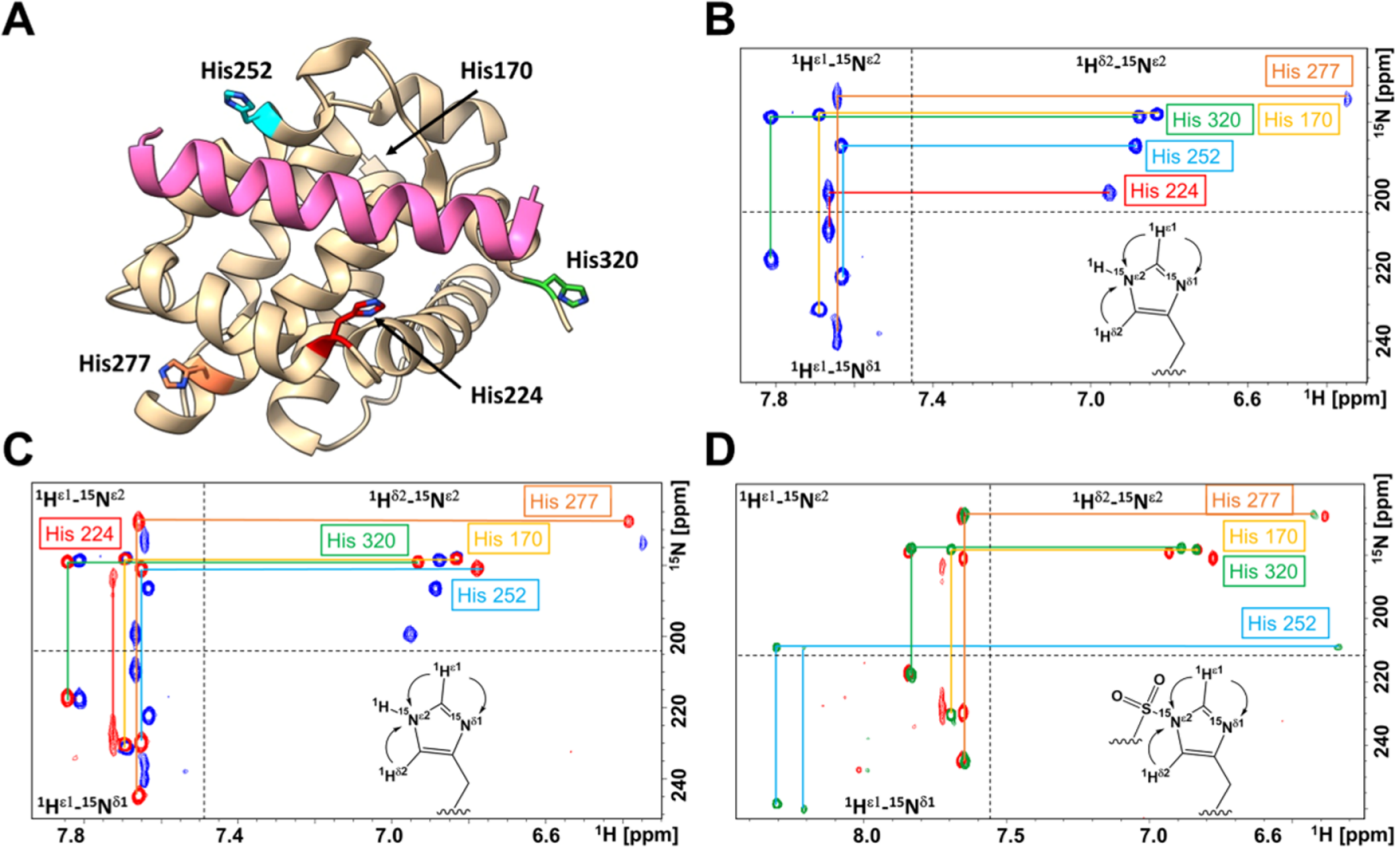
Detection of ligand binding via time-dependent ^1^H and ^13^C^ε^-Met NMR and via measurements of His side chain long-range so-fast HMQC [^15^N,^1^H] correlation spectra. (A) Ribbon representation of wt hMcl-1 (beige) and the BIM BH3 peptide (pink) in the binding pocket. His170, His224, His252, His277, and His320 are also highlighted. (PDB ID 2PQK). (B) His side chain long-range so-fast HMQC [^15^N,^1^H] correlation spectrum of 50 μM *wt* hMcl-1(172–323). Resonance assignments were obtained from the literature^35^ (BMRB ID 19654; bmrb.io) and confirmed with single point mutations (Supporting Figures S21 and S22). (C) His side chain long-range so-fast HMQC [^15^N,^1^H] correlation spectra of 50 μM wt hMcl-1(172–323) in the absence (blue) and in the presence (red) of 250 μM BIM BH3 peptide (Supporting Figure S23 further supports the assignment of the cross-peaks of His224). (D) His side chain long-range so-fast HMQC [^15^N,^1^H] correlation spectra of 50 μM wt hMcl-1(172–323) in the presence of 250 μM BIM BH3 peptide (red) and of 250 μM **155H1** stapled peptide (green) (Supporting Figure S24 further supports the assignment of the cross-peaks of His252).

Further characterizations of the binding of our covalent agents were driven by NMR spectroscopy measurements using 1D ^1^H-aliphatic NMR, 2D [^13^C,^1^H] correlation spectra with a ^13^C^ε^-Met-labeled hMcl-1(172–323), and long-range [^15^N,^1^H] correlation spectra with a uniformly ^15^N-labeled hMcl-1(172–323), focusing on observing the side chain resonances of aliphatic residues, Met, or His, respectively, that are located in the BH3 binding pocket of hMcl-1(172–323) (Figure 5A). Analysis of the 1D ^1^H aliphatic region of the spectrum of hMcl-1(172–323) upon ligand binding indicated specific time-dependent chemical shift perturbations that reflected the binding and covalent adduct formation of the stapled peptide with hMcl-1(172–323) (Figure S19). This was evident, for example, for the binding site ^1^H methyl resonances for residues Thr259 and Ile181 (Figure S19). Similarly, analysis of the 2D [^13^C, ^1^H] correlation spectra with a ^13^C^ε^-Met-labeled hMcl-1(172–323) sample collected in the absence and in the presence of stapled compound **11** resulted in large and time-dependent chemical shift perturbation for binding site Met 250 side chain ^13^C^ε^/^1^H^ε^ resonances (Supporting Figure S19), again suggesting binding of the peptide and subsequent reaction with His 252. The 2D [^1^H,^13^C] correlation spectrum of ^13^C^ε^-Met hMcl-1(172–323) in the apo form is reported in Supporting Figure S20.

The imidazole ring proton and nitrogen resonances for the 5 His residues in uniformly ^15^N-labeled hMcl-1(172–323) (Figure 5A) are clearly detected with a long-range so-fast HMQC spectrum (Figure 5B), and assignments were obtained based on previously reported NMR studies.^35^ Upon binding to the noncovalent BIM peptide (Table 1), resonances of the His 224 imidazole ring were shifted and largely broadened, and smaller but sizable perturbations were also observed for the side chain of His 252 and His 277, while smaller chemical shift changes were observed for the other His residues, located further outside the BH3 binding region (Figure 5C). However, when the protein was exposed to stapled covalent agent **155H1**, resonances of the binding site His 224 further broadened beyond detection, while the cross-peaks for the imidazole resonances of His 252 were dramatically shifted for all 3 observed resonances (Figure 5D), presumably due to the formation of the covalent adduct with His 252. A minor second set of resonances was observed for His 252, which also largely shifted compared to the same spectra measured for both apo hMcl-1(172–323) or BIM bound hMcl-1(172–323), likely representing a slightly different bound conformation (Figure 5D).

### Structural Characterization of Covalent Stapled Peptide 155H1 in Complex with hMcl-1(172–323)

**Figure 6:**
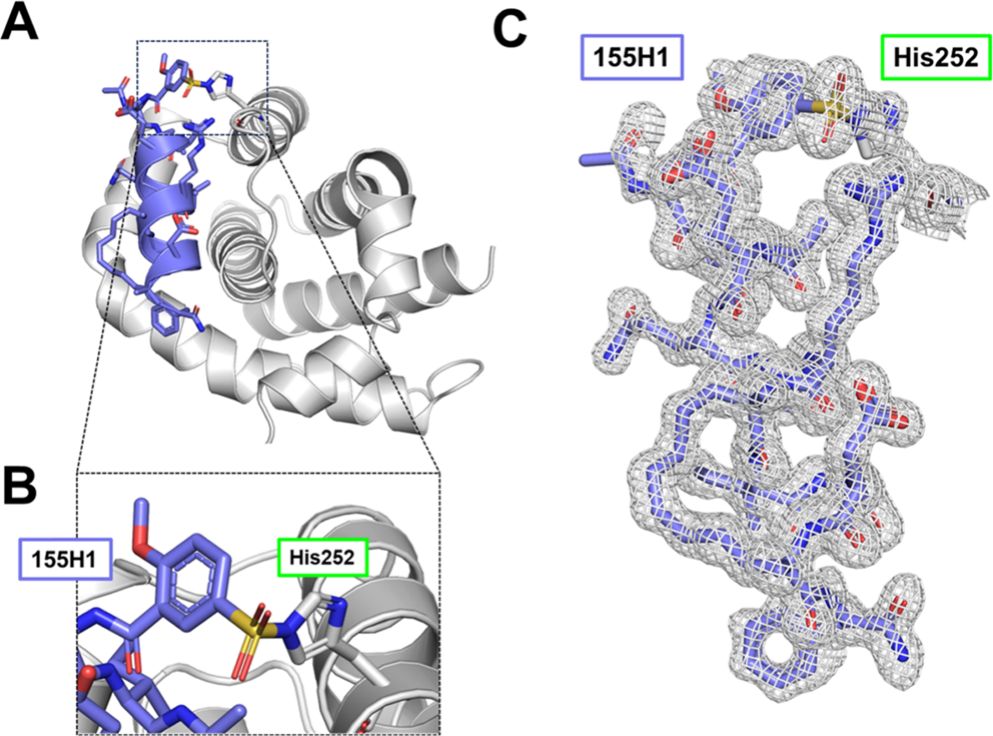
Crystal structure of hMcl-1(172–323) in complex with His-covalent stapled peptide 155H1. (A) Ribbon representation of hMcl-1(172–323) (gray) in complex with **155H1** (blue). The side chains of the agent are also displayed. (B) Close-up view of the covalent sulfonamide bond resulting from the reaction of the sulfonyl fluoride of compound **155H1** and the side chain of His 252. (C) 2Fo–Fc map of bound compound **155H1** contoured at 1.5 sigma.

To further elucidate the binding conformation of the covalent agent to hMcl-1(172–323) and to more unambiguously identify the covalently targeted residue, we determined the X-ray structure of compound **155H1** in complex with hMcl-1(172–323). A summary of the structural parameters is reported in Supporting Table S1. Overall, the geometry of the general structure of the complex is similar to the previously determined structure of hMcl-1(172–323) in complex with BH3 peptides (Supporting Figure S25), with a monomer of hMcl-1(172–323) superimposed with PDB entry 6VBX with a rmsd value of 0.8 Å (Supporting Figure S25). Similarly, the overall position of the BH3 mimetic is typical of such peptides (Supporting Figure S26). As anticipated by previous characterizations, we found that the N-terminal residue formed a covalent bond with the side chain of His 252, as observed by a contiguous electron density (Figures 6C) between the aryl-sulfonyl group and the imidazole ring of His 252 with whom the group formed a stable sulfonamide (Figure 6B). These findings provide further and unconfutable evidence of the previous data with SDS gel electrophoresis, mass spectrometry, and NMR data, all of which strongly supported covalent adduct formation between these two molecules and specifically with His 252. In the final refined model, the density and placement of covalently bound **155H1** in the hMcl-1(172–323) structure were well resolved in its entirety (Figure 6). The general peptide-hMcl-1(172–323) interactions appeared well maintained in the crystal structure, in which the amphipathic helical peptide was inserted into the peptide-binding pocket with its hydrophobic side chains buried in the pocket, while the peptide’s only Asp residue was involved in a salt bridge with hMcl-1(172–323) residue Arg 263. These interactions are typically well conserved between BH3 peptides and hMcl-1(172–323)^12^ as well as in other BH3/Bcl-2 protein complexes.^36^

### Cellular Studies with Stapled Peptides

**Figure 7:**
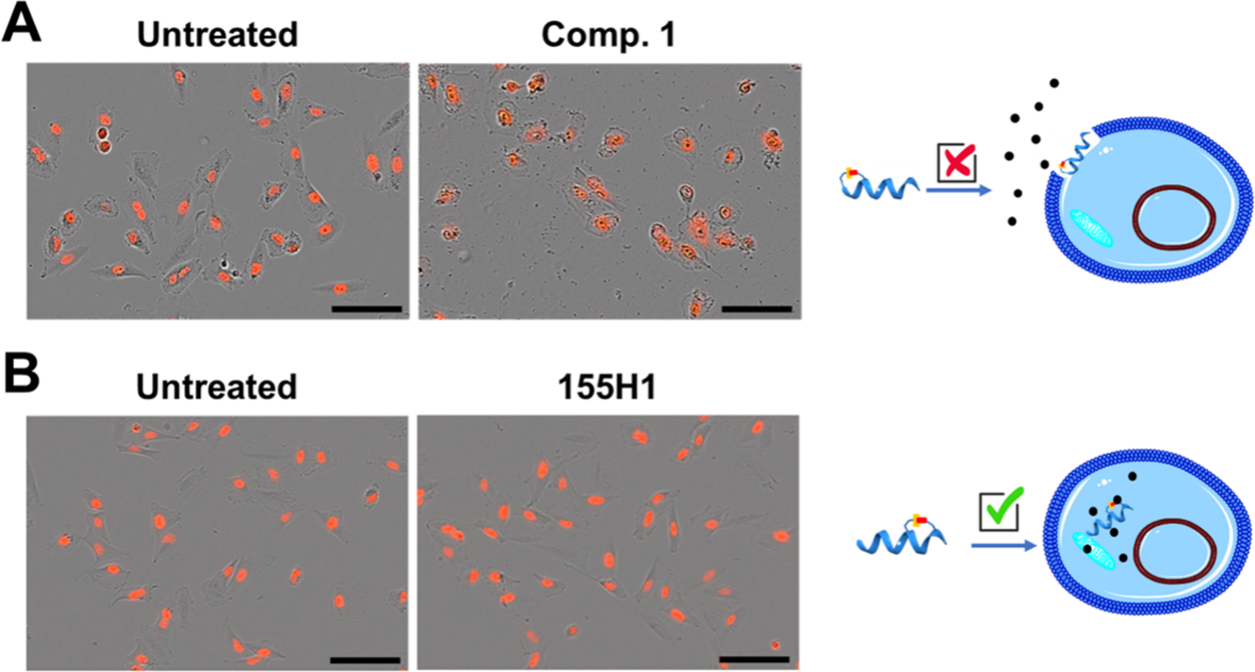
Lytic effect of stapled peptides on the NSCLC A549 NucLight Red cells detected via the IncuCyte S3 live-cell analysis system. The schematic illustrations depict the lytic (panel A) and nonlytic (panel B) effects of stapled agents on the cells. Representative images of cells collected 20 min after exposure to 100 μM lytic stapled compound **1** (panel A) or nonlytic stapled peptide **155H1** (panel B), along with untreated (DMSO only) cells as a control. Note: Images were taken 20 min after plates were placed inside the IncuCyte S3 to minimize condensation that can affect image quality. The scale bar denotes 100 μm.

Amphipathic alpha-helical peptides have been reported to cause nonspecific membrane disruption events due to their constrained helical structure and amino acid composition.^2,5,37,38^ Lytic peptides in fact are highly efficient at binding and disrupting the negatively charged membrane of cells, as they are usually amphipathic and positively charged.^2,5,37,38^ Key determinants for cell permeability and the lytic effect are the combination of relatively high hydrophobic content and the number of positively charged residues in addition to the position of the staple within the alpha-helical peptide. Therefore, our design strategy included peptides with varying net charges and explored different positions of the staple within the peptide (Tables 1 and 2; Figure 2). To assess cellular permeability and to detect eventual nonspecific cell lytic effects, we tested selected stapled covalent peptides against the human nonsmall cell lung cancer (NSCLC) cell line A549 (Figure 7). To enhance live cell analysis, we used a cell line that was fluorescently labeled (A549 NucLight Red cells, Essen Bioscience), and cells were monitored for cell lysis via fluorescence microscopy using the IncuCyte S3 live-cell analysis system (Figure 7). When A549 cells were exposed to our agents, a lytic effect was observed only with a few peptides when tested at high concentrations (specifically with compounds **1** and **2**), as evidenced by nearly immediate cell bursting after the exposure of cells to the peptides (Figure 7). It is not surprising that compounds **1** and **2** displayed lytic effects, possessing the largest net positive charge among the designed agents (Table 2). Considering those data, together with the affinity for hMcl-1(172–323), alpha-helical content (Table 2), and covalent complex formation as detected by SDS gel electrophoresis, mass spectrometry, NMR, and X-ray crystallography, we selected agent **155H1**, which was further tested in preliminary cell permeability and cellular efficacy studies.

**Figure 8:**
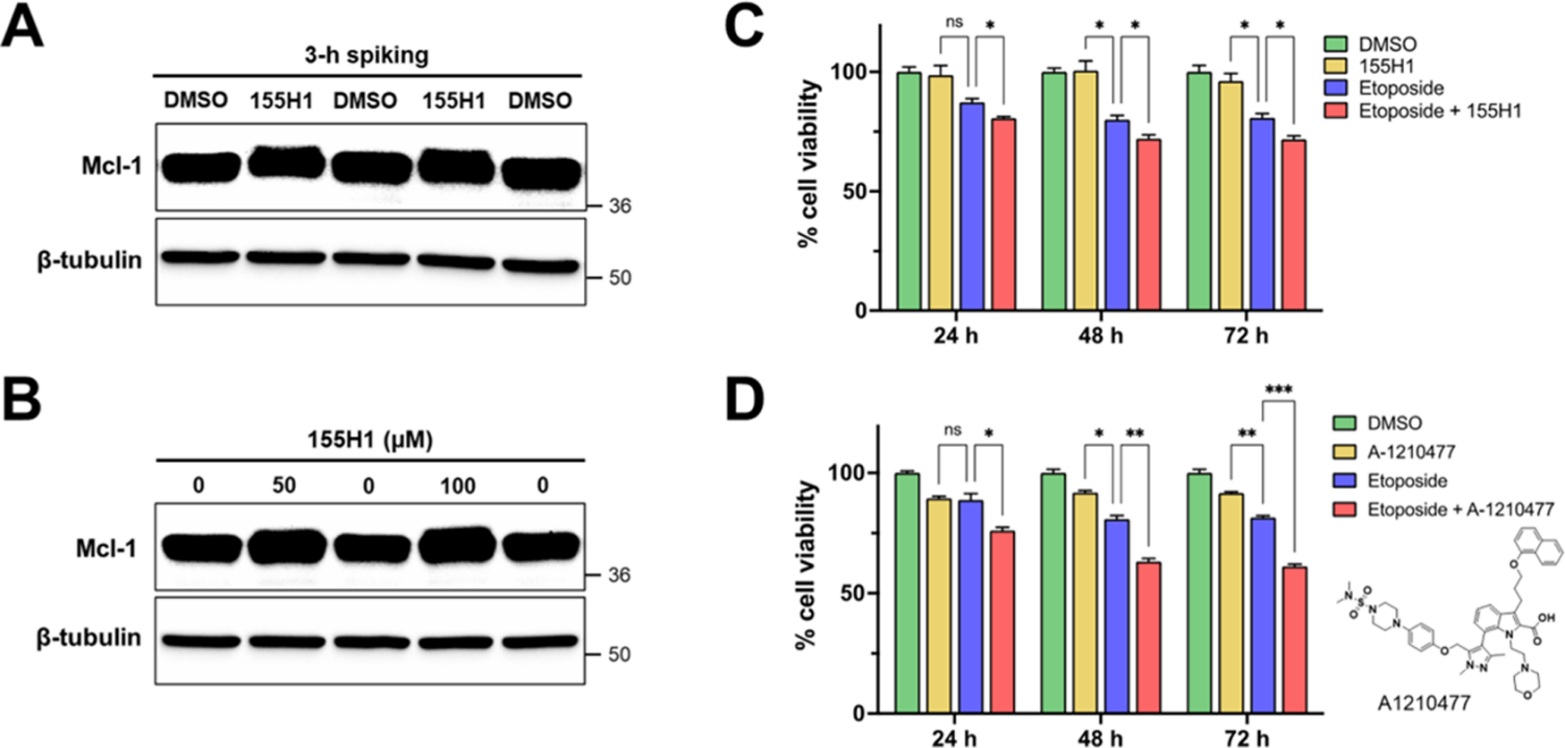
Cell activity of stapled 155H1 peptide in A549 NucLight Red cells. Western blot images of (A) cell lysates spiked with 100 μM 155H1 or 1% DMSO for 3 h at 37 °C and (B) intact cells treated with 1% DMSO, 50 or 100 μM 155H1 for 12 h with the treatments replenished every 3 h. (C) Top: histogram displaying percent cell viability of cells treated with 25 nM etoposide or 50 μM 155H1 either alone or in combination for the indicated times. Bottom as in the top one, but cells were treated with 10 μM small molecule hMcl-1 inhibitor A-1210477. Cell viability was monitored and quantified by the IncuCyte S3 live-cell analysis system. *p < 0.05, as determined by a two-way analysis of variance.

Hence, to assess if the selected His-covalent stapled peptide was able to form a covalent adduct in complex cellular milieus, we lysed A549 cells under nondenaturing lysis conditions (see Experimental Section) and then spiked the lysates with **155H1** prior to running a Western blot (WB) using a specific hMcl-1 antibody (Figure 8A). Under these experimental conditions, **155H1** appeared to form a stable adduct in the cell lysates, as observed by a band shift in the WB (Figure 8A), suggesting that the warhead was stable in the complex cellular milieu. This is consistent with previous studies with the chosen electrophile.^31^ Of note, full-length wt hMcl-1 is much larger than the recombinant construct (hMcl-1(172–323)) used in the SDS gel electrophoresis with Coomassie blue stain, as reported in Figure 4. Nonetheless, the shift in the MW was still evident in the WB (Figure 8A). Likewise, a shift in the band was also appreciable when intact A549 cells were exposed to **155H1** (Figure 8B). These data suggest that **155H1** is potent, His-covalent, nonlytic, and can interact covalently with hMcl-1 in cells. To further assess if this inhibition resulted in sensitization of A549 cells to chemotherapy, a synergy study with etoposide and **155H1** was carried out (Figure 8C). Targeting Mcl-1 per se may not cause cell apoptosis in the absence of a pro-apoptotic stimulus such as etoposide. Accordingly, combination treatment of etoposide and **155H1** led to a modest but significant decrease in cell viability compared to either agent alone, and the observed activity was similar to what was observed with treatment with the potent small molecule hMcl-1 inhibitor A-1210477^39^ (Figure 8C).

Collectively, our data suggest that the selected stapled His-targeting peptides can effectively target hMcl-1 in vitro and in cells. Hence, such agents could find applications in basic or translational studies to evaluate the targeting of hMcl-1 via stapled peptides as a possible therapeutic strategy. Collectively, our data present a novel application for His-covalent strategies in the design of potent and selective stapled alpha-helical peptides that can be generally used to derive novel covalent pharmacological tools or even therapeutics.

## Discussion and Conclusions

The success of covalent drugs targeting Cys in recent years^22,23,40−54^ continues to drive drug discovery efforts toward the *Cysteinome*,^52−54^ which is the target space that contains a *druggable* Cys residue. However, expanding such relatively limited target space to other more frequently occurring residues such as Lys or Tyr is emerging.^27,55−60^ Our recent studies^11,27,31,58^ and others^61,62^ revealed that it is possible to target Lys residues located at protein–protein interfaces, and reports have already emerged on the *Lysinome* and the *Tyrosinome* as ensembles of possible targets that present a “targetable” Lys or Tyr in proximity of their binding sites for cofactors in enzymes.^63,64^ However, studies on the design of ligands targeting His residues remain sparse, despite His is, in principle, more suitable than both Lys and Tyr for covalent modifications.^26^ In a first attempt to assess the reactivity of aryl-fluorosulfates or aryl-sulfonyl fluorides to His residues, we recently reported on an artificial model system where an His residue was inserted by mutagenesis in the binding site of the BIR3 domain of XIAP and its reactivity was assessed with a small library of covalent binders (Smac mimetics).^27^ This preliminary data suggested that His residues can indeed be efficiently targeted by both electrophiles. A more recent example included the incorporation of sulfonyl fluorides^29^ or other electrophiles^30^ to obtain potent covalent ligands of the cereblon E3 ubiquitin ligase complex targeting His 353.^29^

In targeting Mcl-1, previous attempts with covalent agents have been reported, including either a small molecule^61^ or a linear peptide,^12^ with both efforts directed at Lys 234. However, two His residues, namely, His 252 and His 224, are present in the BH3 binding pocket of Mcl-1 (Figures 1 and 5A) and could be therefore targeted perhaps more efficiently with covalent agents compared to Lys 234. Given that His 252 is located across Lys 234 and that we could reach Lys 234 covalently using a linear BH3 peptide,^12^ we sought here to target either Lys 234 or His 252 with modified stapled BH3-derived peptides (Figure 2). We probed various stapled peptides (Figure 2) that were designed based on our previously determined X-ray structure of the complex between hMcl-1(172–323) and a linear BH3 covalent peptide.^12^ As expected, most stapled peptides were found to adopt preferentially an alpha-helical conformation in solution (Table 2) based on CD measurements. Moreover, we found that the peptides reacted efficiently with both wt hMcl-1(172–323) and a Lys234Ala mutant hMcl-1(172–323) (Figure 3), suggesting that preferentially the agents bound to His 252 (Figures 1, 3, and 4), although covalent adduct formation, to a lesser extent (Figure 4C), was also observed for the His252Ala hMcl-1 mutant. While our data speculatively may suggest that the His is more nucleophilic than Lys for covalent modification by the sulfonyl fluoride, other factors including steric, or the residue’s environment, may affect their ability to react with the chosen electrophile. For example, the presence of other basic residues (Lys, Arg, or another His) in the environment of the targeted residue (His or Lys) could enhance their nucleophilicity.^56,65^ However, His 252 and Lys 234 are both solvent-exposed and apparently not involved in any significant intramolecular interactions with Mcl-1. Because the covalent adduct can be formed only when a nucleophilic residue (Lys/His/Tyr) is properly juxtaposed with the ligand, the agents were inactive toward hBfl-1, lacking such residues in a position similar to hMcl-1. This was evident by the DELFIA displacement assay (Figure 3) and by SDS gel electrophoresis (Figure 4D). The latter data further corroborated that the agents were selective in targeting hMcl-1, despite the presence of the attenuated sulfonyl fluoride electrophile,^31^ given that the hBfl-1(1–142) construct used in the experiment contained 7 His, 10 Lys, and 5 Tyr residues.

NMR spectroscopy measurements, particularly those designed for the detection of the His imidazole side chain ^15^N–^1^H resonances, also revealed that His 252 resonances were dramatically affected by the binding of our stapled covalent agent (Figures 5E and S24). Of note, His side chain ^15^N–^1^H correlation spectra were revealed to be very sensitive and informative, with a very large chemical shift dispersion and chemical shift perturbations upon complex formation (Figure 5C–E) and likely could be deployed in covalent fragment screening campaigns aimed at targeting His residues. Finally, the unambiguous identification of His 252 as the targeted residue by the covalent agents was provided by the X-ray structure of the complex (Figure 6), in which contiguous electron density between **155H1** and hMcl-1(172–323) was observed, which was compatible with the formation of a sulfonamide bond between the peptide and the imidazole side chain of His 252 (Figure 6). Preliminary cellular studies also suggest that a covalent adduct can be formed in the complex cellular milieu in NSCLC A549 cells, again attesting that the electrophile presented a proper balance of general stability in the unbound state^31^ and reactivity when properly juxtaposed to the targeted His residue. The fact that the agent can also interact covalently with Lys 234 in the absence of His 252 may also provide an advantage to possible resistance by mutation mechanisms.

Overall, our studies clearly suggest that His-covalent strategies can be incorporated into any ligand optimization approaches, addressing potency and selectivity without compromising other properties of the resulting agents. This is particularly significant because His is frequently occurring in binding sites of drug targets, including enzyme catalytic sites,^66^ and perhaps most importantly, it is frequently found to be in close proximity to drugs and binding sites,^28^ possibly providing an immediate covalent optimization strategy for a large number of reversible ligands.^26^ However, further examples of His-targeting agents will need to emerge in the near future to substantiate our enthusiasm for the approach.

In conclusion, our studies support the vision that strategies targeting covalently His residues should be incorporated into drug discovery and lead optimization campaigns that have emerged in the past decade, including fragment-, structure-, and/or NMR-based approaches,^8,67−72^ and DNA-encoded libraries,^64,73,74^ aimed at deriving novel, potent, selective, covalent agents for continued drug development.

## Experimental Section

### General Chemistry

For the synthesis of the reported agents, we followed standard solid-phase strategies using a Rink amide resin. Coupling reactions were performed manually or with the use of an automated Liberty Blue Peptide Synthesizer (CEM Corp.). All reagents were commercially available, including Fmoc (fluorenylmethyloxycarbonyl)-protected amino acids, the N-terminal acid to obtain the electrophile, and resins that were used without further purification. The synthetic protocol for each coupling that was performed manually involved 3 equiv of Fmoc-amino acid, 3 equiv of *O*-(7-azabenzotriazol-1-yl)-*N*,*N*,*N*′,*N*′,-tetramethyluronium hexafluorophosphate (HATU), 3 equiv of OximaPure, and 5 equiv of *N*,*N*-diisopropylethylamine (DIPEA) in 1 mL of DMF (dimethylformamide) (r.t., 1 h). The protocol for the coupling with the Liberty Blue Synthesizer involved 6 equiv of Fmoc-amino acid, 3 equiv of *N*,*N*′-diisopropylcarbodiimide (DIC), and 1 equiv of OximaPure in 2 mL of DMF (90 °C, 5 min via microwave irradiation). Fmoc deprotection was performed by treatment with 20% 4-methylpiperidine in DMF (2 × 1 mL for 5 and 20 min if performed manually; 2 × 3 mL, 90 °C for 3 min if performed with the Liberty Blue Synthesizer). Prior to the final peptide cleavage, an on-resin ring-closing metathesis reaction was performed to build the staples. In agent compound **9**, the staple was created with a click reaction, which was performed in solution after the cleavage of the peptide. Peptide cleavage from the resin was accomplished with a mixture containing TFA (trifluoroacetic acid), triisopropylsilane, water, and phenol (94:2:2:2) for 5 h, followed by precipitation of the peptide in cold diethyl ether. After redissolving the precipitate agents in DMSO, each solution was purified by preparative RP-HPLC using an XTerra C18 column (Waters) with a JASCO preparative HPLC system and gradient water/acetonitrile (5–100%) containing 0.1% TFA (purity > 95%). The identity of the peptides was further confirmed by high-resolution mass spectrometry.

#### On-Resin Ring-Closing Metathesis (RCM)

To prepare for RCM, the resin was washed with DCM and dried thoroughly under a high vacuum. Next, the dry resin was swollen in dry dichloroethane (DCE) under a stream of N_2_ for 10 min and drained. The RCM reaction was performed by treating the resin with a 10 mM solution of Grubbs catalyst first generation in dry DCE (2 mL per 50 μmol of resin) under N_2_ for 2 h. The resin was then drained of the catalyst solution, and a fresh 10 mM Grubbs catalyst solution was added to it and reacted for 2 more hours. Finally, the resin was washed with DCM and DMF (Supporting Figure S1).

#### In-Solution Click Reaction

The purified peptide, containing an azide and an alkyne, was dissolved in 2.4 mL of DMSO. 0.5 mL portion of MQ Water, 50 μL of Na-ascorbate solution 1 M, and 50 μL of CuSO_4_ solution 1 M were added. The reaction was performed overnight, and the final product was purified by preparative RP-HPLC (Supporting Figure S2).

#### Compound Purification

RP-HPLC purification was performed on a JASCO preparative system equipped with a PDA detector and a fraction collector controlled by a ChromNAV system (JASCO) on an XTerra C18 10 μm 10 × 250 mm^2^ (Waters). The purity of tested compounds was assessed by HPLC (purity > 95%). **155H1** purity analysis is reported in Figure S27 (purity of >98%).

#### Protein Expression and Purification

A cDNA fragment encoding the ligand-binding domain of hMcl-1 (residues 172–323) was cloned into a pET15b vector with an N-terminal His tag and a thrombin cleavage site. This vector was used to express both hMcl-1(172–323) and methyl-^13^C^ε^-methionine-labeled hMcl-1(172–323). The plasmid was transformed into BL21 (DE3) gold pLysS competent cells, which were grown in an LB medium at 37 °C with 100 μg/mL ampicillin. Once the cell density reached OD600 of 0.6–0.7, 1 mM IPTG was added to induce expression, and the cells were allowed to grow overnight at 20 °C. The bacteria were then collected by centrifugation. To obtain ^13^C^ε^-methionine hMcl-1(172–323), 100 mg of ^13^C^ε^-l-methionine, suspended in 1 mL of DMSO, was added per liter of an LB medium 10 min before induction. The same protocol had been used for the expression of protein wt hBfl-1. A cDNA fragment encoding residues 1–149 of hBfl-1 was cloned into a modified pET21a vector with an N-terminal His tag and a TEV cleavage site. The BL21 (DE3) gold pLysS competent cells were used for the transformation, and they were grown in an LB medium at 37 °C with 50 μg/mL Kanamycin. Once the cell density reached OD600 of 0.6–0.7, 0.1 mM IPTG was added to induce expression, and the cells were allowed to grow overnight at 15 °C before collection by centrifugation.

The collected bacteria were lysed by sonication, and the overexpressed proteins containing an N-terminal His tag were purified using immobilized metal ion affinity chromatography (IMAC) with a linear gradient of imidazole (elution buffer: 25 mM Tris at pH 7.5, 500 mM NaCl, and 500 mM imidazole). Finally, the protein was further purified and buffer exchanged through size-exclusion chromatography with a HiLoad 26/60 Superdex 75 preparative-grade column into an aqueous buffer composed of 50 mM phosphate at pH 7.5, 150 mM NaCl, and 1 mM DTT.

### Circular Dichroism

Circular dichroism spectra were acquired on a Jasco 815 spectropolarimeter. Samples were prepared in MQ Water or in 15 mM phosphate buffer, pH 7.5. The experiments were conducted at 25 °C, using a 1 mm-path length quartz cell to record data from 190 to 260 nm in 1 nm increments. The response time was 2 s. The data were then converted to Δε(M^–1^·cm^–1^), and the percentage of alpha-helix was calculated using BeStSel (https://bestsel.elte.hu) software.^34^

#### SDS Gel Electrophoresis

SDS-poly(acrylamide gel electrophoresis) (PAGE) was performed by incubating either *wt* hMcl-1(172–323), hMcl-1(172–323) K234A, or wt hBfl-1(1–149) at 10 μM concentration in a buffer 25 mM phosphate pH 7.5, 150 mM NaCl with or without each agent at the concentration of 100 μM. Each protein was incubated with each peptide for 16 h at room temperature. Next, the samples were subjected to gel electrophoresis using SDS-PAGE with the NuPAGE 12% Bis-Tris gels (Life Technologies) and MOPS as the running buffer. Finally, the samples were stained with Simply Blue Safe Stain (Life Technologies) following the manufacturer’s instructions.

### NMR Spectroscopy

NMR spectra were acquired on a Bruker Avance III 700 MHz spectrometer equipped with a TCI cryoprobe. All NMR data were processed and analyzed using TopSpin 4.1.0 (Bruker, Billerica, MA). 1D ^1^H-aliph and 2D-[^13^C,^1^H]-HSQC experiments were performed with a 20 μM hMcl-1(172–323) sample or a ^13^C^ε^-Met hMcl-1(172–323) sample (in 50 mM phosphate pH = 7.5, 150 mM NaCl, and 1 mM DTT) in the absence or in the presence of 100 μM selected peptides, and the experiments were acquired at various time points. 2D-[^15^N,^1^H] long-range so-fast HMQC spectra were optimized to detect His side chain ^2^*J*^15^N–^1^H correlations. Spectra were collected with samples of 50 μM ^15^N hMcl-1(172–323) (in 50 mM phosphate pH = 7.5, 150 mM NaCl, and 1 mM DTT) in the absence or in the presence of 250 μM BIM BH3 peptide or His-covalent stapled peptide **155H1**.

### Biochemical DELFIA Assay

A solution containing 600 ng/mL of biotinylated-BH3 peptide (biotin-aminohexanoic acid-EDIIRNIARHLAQVGDSMDR-NH_2_) was added to each well of 96-well streptavidin-coated plates (PerkinElmer). At the end of a 2 h incubation, plates were washed 3 times. Subsequently, a solution of 6His-tagged-hMcl-1(172–323) or 6His-tagged-hBfl-1(1–149) protein and the test peptides were preincubated for 2 h and added to the washed streptavidin-coated plates along with a Eu–N1-labeled anti-6xHis antibody (PerkinElmer, 1:2000) solution and further incubated for 2 h on a microplate shaker. Plates were then washed 3 times, and each well was incubated with 200 μL of the enhancement solution (PerkinElmer) for 10 min. Fluorescent readings (VICTOR X5 microplate reader) were then measured by using the excitation and emission wavelengths of 340 and 615 nm, respectively. Fluorescent counts were normalized to those of DMSO wells and reported as % inhibition, and Prism 10 (GraphPad) was used to calculate the IC_50_ values. Protein, peptide, and antibody solutions were prepared in a DELFIA assay buffer (PerkinElmer), and all of the incubations were performed at room temperature. Each well received a final DMSO concentration of 1%. The final concentrations of 6His-tagged-hMcl-1(172–323) and 6His-tagged-hBfl-1(1–149) used were 16 and 15 nM, respectively.

### Cell Lines, Cell Culture, and Antibodies

Nonsmall cell lung cancer (NSCLC) A549 NucLight Red cells were purchased from Essen Bioscience and cultured in Ham’s F-12 nutrient mixture with GlutaMAX-1 (Gibco) supplemented with 10% FBS and 0.5 μg/mL puromycin. Cells were maintained in a humidified incubator at 37 °C in 5% CO_2_. These cells have nuclei-fluorescently labeled red. Anti-hMcl-1 and anti-β-tubulin antibodies were purchased from Cell Signaling Technology (#5453) and Santa Cruz Biotechnology (sc-58884), respectively. HRP-conjugated goat antimouse and antirabbit secondary antibodies were purchased from ThermoFisher Scientific.

### Immunoblotting Assays

Cells were treated with 50 or 100 μM compounds or 1% DMSO for 12 h and lysed with a lysis buffer (20 mM Tris, pH 7.4, 120 mM NaCl, 1% Triton X-100, 0.5% sodium deoxycholate, 0.1% SDS, 1% IGEPAL, and 5 mM EDTA, supplemented with EDTA-free Protease Inhibitor Cocktail and PhosStop (Sigma-Aldrich)) for 15 min on ice. Cell lysates were then centrifuged to clear off cellular debris for 20 min at 13 000 rpm at 4 °C, and supernatants were collected. In the case of spiking the cell lysates, 100 μM compounds were added to the supernatants, and the mixture was incubated for 3 h at 37 °C. Samples were prepared and loaded into 12% NuPAGE Bis-Tris Precast Gels and transferred onto PVDF membranes. The membranes were then blocked with 5% nonfat milk in TBS and 0.1% Tween (TBST) for 1 h, probed for primary antibodies raised against hMcl-1 or β-tubulin at 4 °C overnight, and then with secondary antibodies for 1 h. Subsequently, Clarity Western ECL solution (BIO-RAD) was added to the blots prior to being imaged on a FluorChem (ProteinSimple) instrument and analyzed with ImageJ software.

#### Cell Viability Assays

A549 NucLight Red cells were seeded at 5000 cells per well in 96-well plates. Next day, cells were treated with 25 nM etoposide either by itself or in combination with 50 μM **155H1** and imaged with the IncuCyte S3 live-cell analysis system every 3 h for 3 days. The integrated IncuCyte software was used to quantify the number of fluorescently labeled red nuclei. Percent cell viability was calculated by normalizing the red nuclei count of samples to those of DMSO controls.

#### X-ray Crystallography

Crystallization was conducted using sitting drop vapor diffusion at 4 °C, with diffraction quality crystals grown in 0.1 M Imidazole, pH 8.0, and 10% (w/v) PEG 8000. The 10.3 mg/mL hMcl-1(172–323)/**155H1** complex was plated at 0.5:0.5 μL ratio (protein/mother liquor). Crystals were frozen in 20% ethylene glycol cryoprotectant, and data collection was conducted at the Diamond Light Source (I04) beamline. A data set was collected on a crystal that diffracted to 1.1 Å, and the diffraction data was processed in the P212121 space group using XIA2 Dials. Molecular replacement for the data set was performed using a search model based on PDB ID: 6VBX in PHASER, PHENIX.^75^ The top solution was refined using rigid body refinement in REFMAC. Several rounds of refinement and model building were performed in the absence of peptides using COOT and PHENIX. After the placement of the solvent molecules, the chemical model of the noncanonical N-terminal amino acid was fit into the remaining density and refined by PHENIX. The remaining parts of the peptide were built manually using COOT and refined in the structure by PHENIX. The final crystal data statistics are listed in Supporting Table S1. The coordinates for the complex between **155H1** and hMcl-1(172–323) have been deposited in the PDB and will be released upon publication (PDB ID: 8VJP).

## Supporting Information Available

Reports a summary of structural parameters for the crystal structure of hMcl-1(172–323) in complex with peptide 155H1 (Table S1); reports mass-spectrometry data of stapled peptides (Table S2); illustrates the synthesis of stapled peptide 1 (Figure S1); describes the synthesis of stapled peptide 9 (Figure S2); displays the CD curves of linear peptide 138E12 (Figure S3); display the CD curves of stapled peptides (Figures S4–S14); reports the dose–response DELFIA displacement assay curves for noncovalent analog of stapled peptide 155H1 (Figure S15); displays the time-course for the reaction between 155H1 and Mcl-1(172–323) using SDS-Gel electrophoresis and MS analyses (Figure S16); reports the mass analysis of wt hMcl-1(172–323) (Figure S17); reports the mass analysis of hMcl-1(172–323) K234A (Figure S18); reports the 1D ^1^H and 2D [^1^H,^13^C] correlation spectra of 20 μM ^13^C^e^-Met hMcl-1(172–323) in the presence of compound 11 (Figure S19); reports the 2D [^1^H,^13^C] correlation spectrum of 20 μM ^13^C^e^-Met hMcl-1(172–323) (Figure S20); reports the His side chain long-range so-fast HMQC [^15^N,^1^H] correlations spectra of 50 μM wt hMcl-1(172–323) (blue) and of 50 μM wt hMcl-1(172–323) H252A (red) (Figure S21); reports the His side chain long-range so-fast HMQC [^15^N,^1^H] correlation spectra of 50 μM wt hMcl-1(172–323) (blue) and of 50 μM wt hMcl-1(172–323) H224A (red) (Figure S22); reports the His side chain long-range so-fast HMQC [^15^N,^1^H] correlations spectra of 50 μM wt hMcl-1(172–323) H252A in the absence (blue) and in the presence (red) of 250 μM BIM BH3 peptide (Figure S23); reports the His side chain long-range so-fast HMQC [^15^N,^1^H] correlation spectra of 50 μM wt hMcl-1(172–323) H252A in the absence (blue) and in the presence (red) of 250 μM 155H1 stapled peptide (Figure S24); illustrates the superposition of the X-ray structures of hMcl-1(172–323) covalently bound to 138E12 (PDB ID 6VBX) and to compound 155H1 (PDB ID 8VJP) (Figure S25); illustrates the superposition of the X-ray structures of hMcl-1(172–323) covalently bound to 138E12 (PDB ID 6VBX) or to 155H1 (PDB ID 8VJP) (Figure S26); reports the HPLC trace for compound 155H1 (Figure S27); reports uncropped Western blot images (Figure S28) (PDF)
String formula file (CSV)

## Notes

**PDB ID Code** The atomic coordinates of the model between hMcl-1(172–323) and **155H1** have been submitted to the protein data bank (PDB ID Code 8VJP). Authors will release the atomic coordinates and experimental data upon article publication.

## Abbreviations Used

CD: circular dichroism
CuSO_4_: cupric sulfate
DBU: 1,8-diazabicyclo[5.4.0]undec-7-ene
DCM: dichloromethane
DCE: 1,2-dichloroethane
DELFIA: dissociation-enhanced lanthanide fluorescent immunoassay
DIPEA: *N*,*N*-diisopropylethylamine
DMF: dimethylformamide
DMSO: dimethyl sulfoxide
DTT: dithiothreitol
EDTA: ethylenediaminetetraacetic acid
HATU: 1-[bis(dimethylamino)methylene]-1*H*-1,2,3-triazolo[4,5-*b*]pyridinium 3-oxid hexafluorophosphate
HMQC: heteronuclear multiple quantum coherence
HPLC: high-performance liquid chromatography
IPTG: isopropyl b-d-1-thiogalactopyranoside
LC-MS: liquid chromatography–mass spectrometry
OXYMA: ethyl cyanoglyoxylate-2-oxime
TFA: trifluoroacetic acid
THF: tetrahydrofuran
