# Supplementary material for: Histidine-Covalent Stapled Alpha-Helical Peptides Targeting hMcl-1
Source: J Med Chem. 2024 May 2;67(10):8172–85. doi: 10.1021/acs.jmedchem.4c00277 (PMC11129181; doi:10.1021/acs.jmedchem.4c00277)

Histidine-covalent stapled alpha-helical peptides targeting hMcl-1

Supplemental Information

Giulia Alboreggia,[1] Parima Udompholkul[1], Carlo Baggio[1], Kendall Muzzarelli[2], Zahra Assar[2],

and Maurizio Pellecchia[1]*

[1]*Division of Biomedical Sciences, School of Medicine, University of California Riverside, 900 University Avenue, Riverside, CA 92521, USA.*

[2] *Cayman Chemical Co., 1180 E. Ellsworth rd, Ann Arbor, MI 48108.*

*Corresponding author: Maurizio Pellecchia, phone number: (951) 827-7829; email address: maurizio.pellecchia@ucr.edu

SI content

Table S1 page S4-S5
Summary of structural parameters for the crystal structure of hMcl-1(172-323) in complex with peptide **155H1**.

Table S2 page S6
Mass-spectrometry data of stapled peptides.

Figure S1 page S7
Schematic representation of the synthesis of stapled compound **1**.

Figure S2 page S8
Schematic representation of the synthesis of stapled compound **9**.

Figure S3 page S9
CD curves of linear peptide **138E12**.

Figure S4-S14 page S10-S15
CD curves of stapled peptides.

Figure S15 page S16
Dose–response DELFIA displacement assay curves comparing the ability of non-covalent analog of stapled peptide **155H1**, to displace the binding of a biotinylated-BH3 peptide to hMcl-1(172-

323) (green) or hBfl-1(1-149) (blue). The structure of the non-covalent analog of stapled peptide **155H1** is also reported.

Figure S16 page S17- S19
Time-course for the reaction between **155H1** and Mcl-1(172-323) using SDS-Gel electrophoresis and MS analyses.

Figure S17 page S20
Mass analysis of *wt* hMcl-1(172-323).

Figure S18 page S21
Mass analysis of hMcl-1(172-323) K234A.

Figure S19 page S22
Detection of ligand binding via time-dependent ^{1}H and $^{13}C^{\varepsilon}$-Met NMR measurement. 1D ^{1}H and 2D [^{1}H,^{13}C] correlation spectra of 20 μM $^{13}C^{\varepsilon}$-Met hMcl-1(172-323) in the presence of compound **11**.

Figure S20 page S23
2D [^{1}H,^{13}C] correlation spectrum of 20 μM $^{13}C^{\varepsilon}$-Met hMcl-1(172-323).

Figure S21 page S24
His side chain long range so-fast HMQC [^{15}N,^{1}H] correlations spectra of 50μM *wt* hMcl-1(172-323) (blue) and of 50μM *wt* hMcl-1(172-323) H252A (red).

Figure S22 page S25
His side chain long range so-fast HMQC [^{15}N,^{1}H] correlations spectra of 50μM *wt* hMcl-1(172-323) (blue) and of 50μM *wt* hMcl-1(172-323) H224A (red).

Figure S23 page S26
His side chain long range so-fast HMQC [^{15}N,^{1}H] correlations spectra of 50μM *wt* hMcl-1(172-323) H252A in absence (blue) and in presence (red) of 250μM BIM BH3 peptide.

Figure S24 page S27
His side chain long range so-fast HMQC [^{15}N,^{1}H] correlations spectra of 50μM *wt* hMcl-1(172-323) H252A in absence (blue) and in presence (red) of 250μM **155H1** stapled peptide.

Figure S25 page S28
Superposition of the X-ray structures of hMcl-1(172-323) covalently bound to **138E12** (PDB ID 6VBX) and to compound **155H1** (PDB ID 8VJP).

Figure S26 page S29
Superposition of the X-ray structures of hMcl-1(172-323) covalently bound to **138E12**, represented in pink (PDB ID 6VBX) and to compound **155H1**, represented in purple (PDB ID 8VJP). A close-up view of the covalent sulfonamide bond resulting from the reaction of the

sulfonyl fluoride of **138E12** and **155H1** and the side chain of Lys234 and His252, respectively, are also shown (Panel B).

Figure S27 page S30
HPLC trace for **155H1** (purity >98 %).

Figure S28 page S31
Uncropped western blot images.

Table S1. Summary of structural parameters for the crystal structure of hMcl-1(172-323) in complex with peptide **155H1**. Data collection and refinement statistics.

	**MCL-1/155H1**
**Wavelength**	0.9537
**Resolution range**	31.41 - 1.13 (1.171 - 1.13)
**Space group**	P 21 21 21
**Unit cell**	31.0387 62.8114 81.2239 90 90 90
**Total reflections**	663090 (19736)
**Unique reflections**	55764 (3405)
**Multiplicity**	11.9 (5.8)
**Completeness (%)**	91.13 (49.80)
**Mean I/sigma(I)**	11.11 (0.58)
**Wilson B-factor**	15.34
**R-merge**	0.08323 (1.388)
**R-meas**	0.08679 (1.521)
**R-pim**	0.02417 (0.5958)
**CC1/2**	0.997 (0.375)
**CC***	0.999 (0.738)
**Reflections used in refinement**	55003 (2950)
**Reflections used for R-free**	1986 (121)
**R-work**	0.1900 (0.2946)

**R-free**	0.2202 (0.3202)
**CC(work)**	0.953 (0.699)
**CC(free)**	0.953 (0.626)
**Number of non-hydrogen atoms**	1572
**macromolecules**	1225
**ligands**	122
**solvent**	225
**Protein residues**	152
**RMS(bonds)**	0.011
**RMS(angles)**	1.48
**Ramachandran favored (%)**	97.33
**Ramachandran allowed (%)**	2.67
**Ramachandran outliers (%)**	0.00
**Rotamer outliers (%)**	0.00
**Clashscore**	8.54
**Average B-factor**	22.95
**macromolecules**	21.54
**ligands**	18.90
**solvent**	32.81

Statistics for the highest-resolution shell are shown in parentheses.

Table S2. Mass-spectrometry data of stapled peptides. All the compounds were analyzed using an Agilent 6545 QTOF LC/MS instrument.

ID	Calcd [M]	Obs. (m/z)
Comp.1	1937.0105	$[M+H]^+$ = 1937.91<br>$[M/2+H]^+$ = 970.0136<br>$[M/3+H]^+$ = 647.0117
Comp.2	1961.0330	$[M/2+H]^+$ = 981.5248<br>$[M/3+H]^+$ = 654.6846
Comp.3	1851.9466	$[M+H]^+$ = 1853.9606<br>$[M/2+H]^+$ = 926.9776
Comp.4	1766.8826	$[M+H]^+$ = 1767.8964<br>$[M+Na]^+$ = 1789.8718<br>$[M/2+H]^+$ = 884.9486<br>$[M/2+Na]^+$ = 906.4304
Comp.5	1895.9364	$[M+H]^+$ = 1897.9472<br>$[M/2+H]^+$ = 949.4784
Comp.6	1780.9094	$[M+H]^+$ = 1782.9223<br>$[M/2+H]^+$ = 891.4641
Comp.7	1695.8454	$[M+H]^+$ = 1696.8544<br>$[M+Na]^+$ = 1719.8356<br>$[M/2+H]^+$ = 848.9299
Comp.8	1768.8731	$[M/2+H]^+$ = 885.4417
Comp.9	1807.8652	$[M+H]^+$ = 1808.9018<br>$[M/2+H]^+$ = 904.9573<br>$[M/3+H]^+$ = 603.6372
Comp.10	1838.9149	$[M/2+H]^+$ = 920.9645
Comp.11 (155H1)	1753.8509	$[M+H]^+$ = 1754.8582<br>$[M/2+H]^+$ = 877.9323

Figure S1. Schematic representation of the synthesis of stapled Compound **1** and mechanism of Grubbs Metathesis reaction.

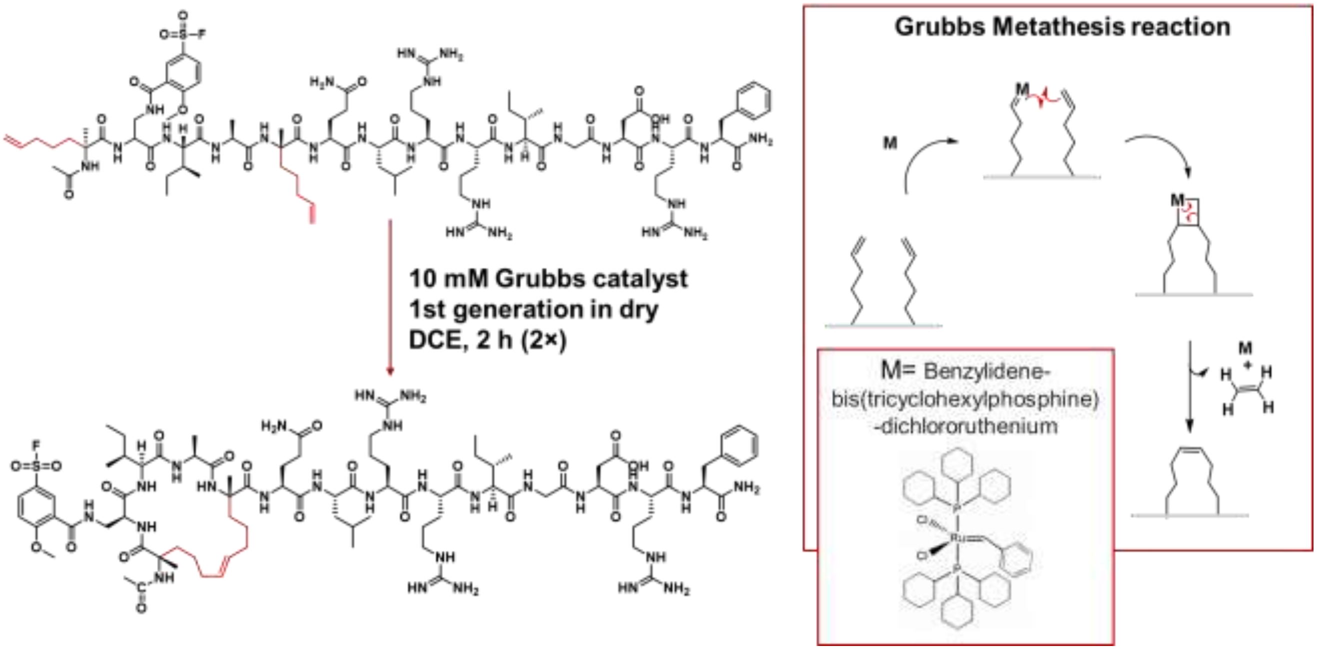

Figure S2. Schematic representation of the synthesis of stapled Compound **9** and mechanism of Click reaction.

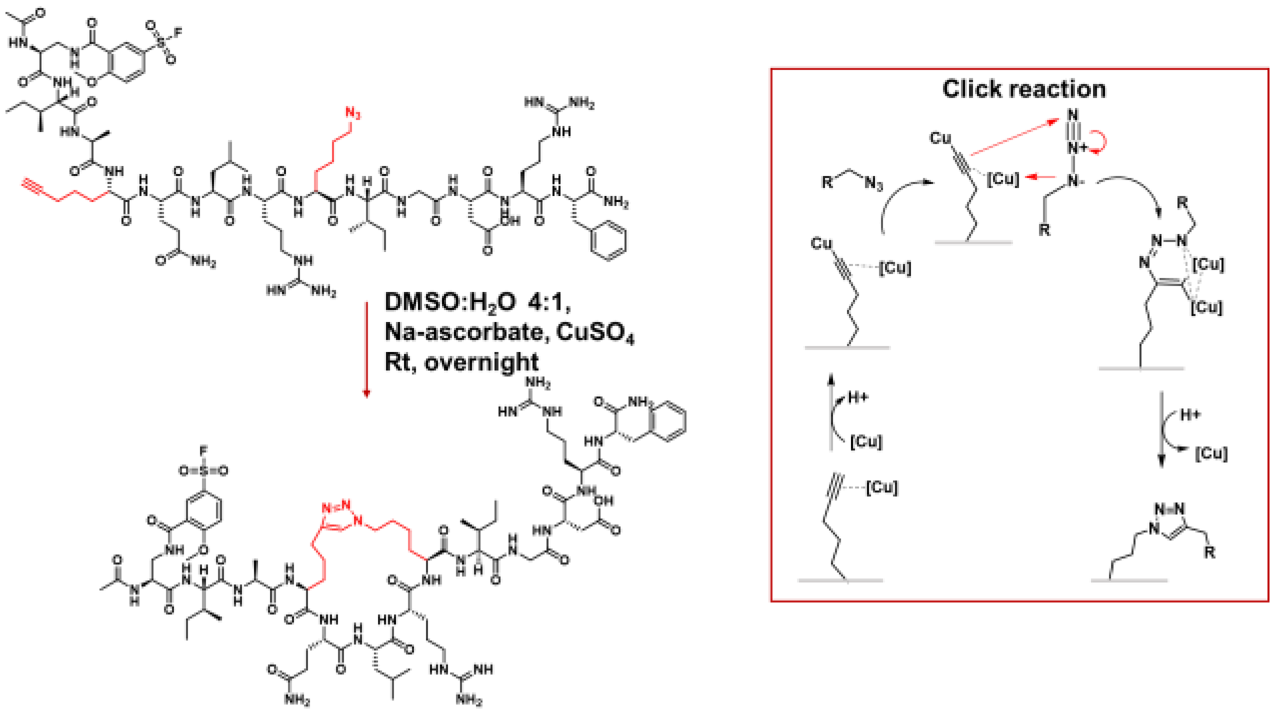

Figure S3. CD curves of linear peptide **138E12** in pure water (blue) and in 15 mM Phosphate buffer pH = 7.5 (red)

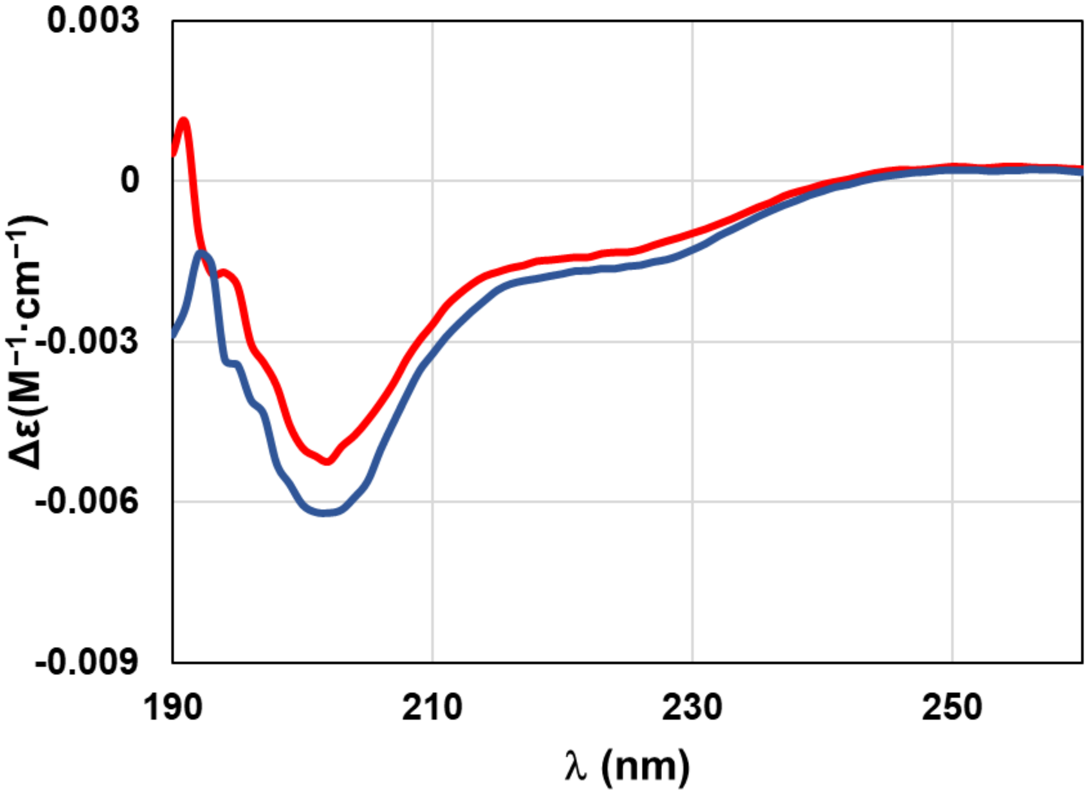

Figure S4. CD curve of stapled Compound **1** in MQ water.

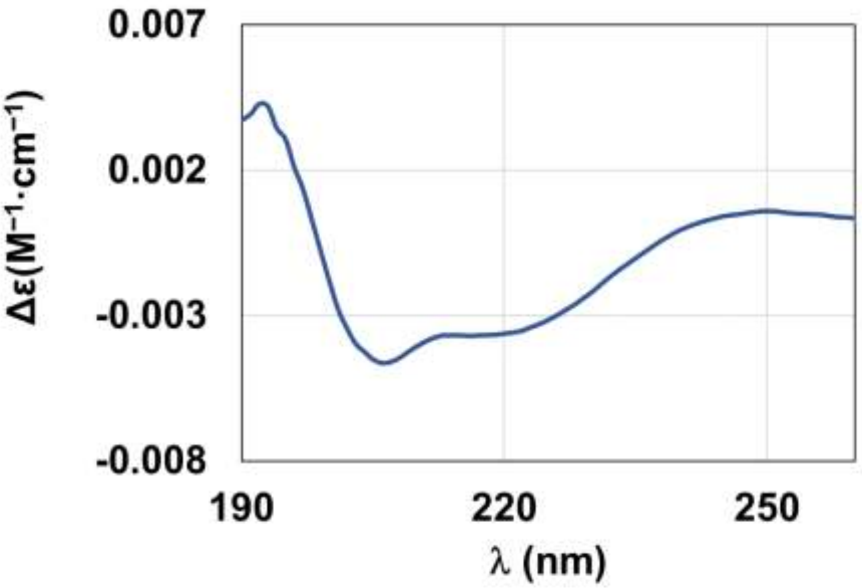

Figure S5. CD curve of stapled Compound **2** in MQ water.

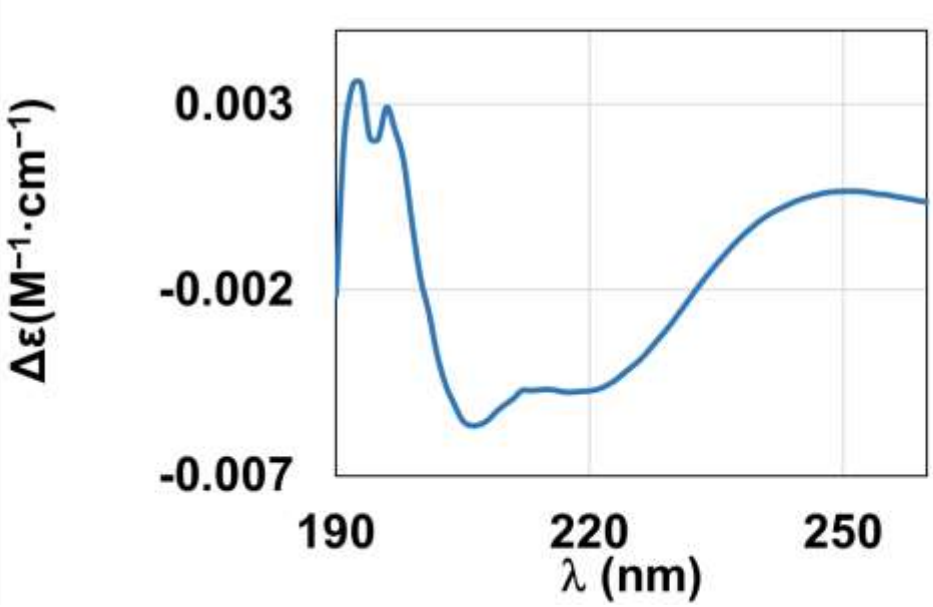

Figure S6. CD curve of stapled peptide Compound **3** in MQ water.

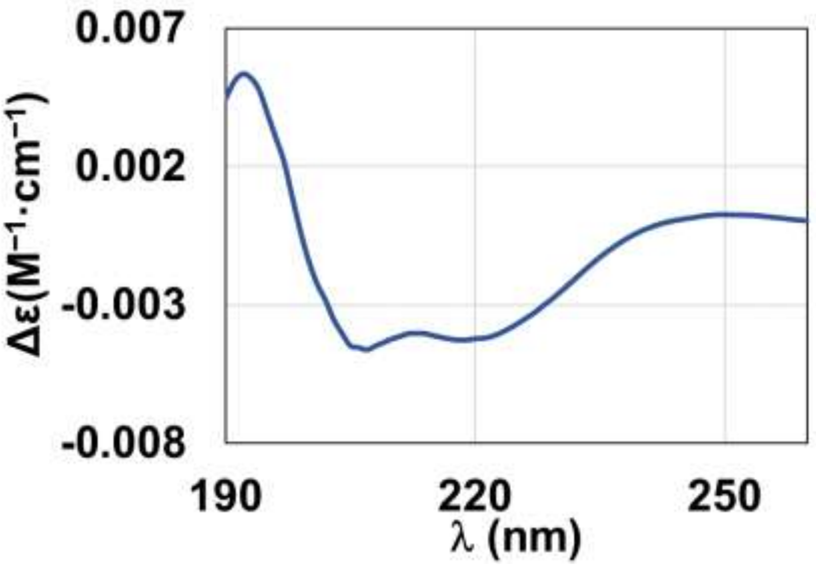

Figure S7. CD curve of stapled peptide Compound **4** in MQ water.

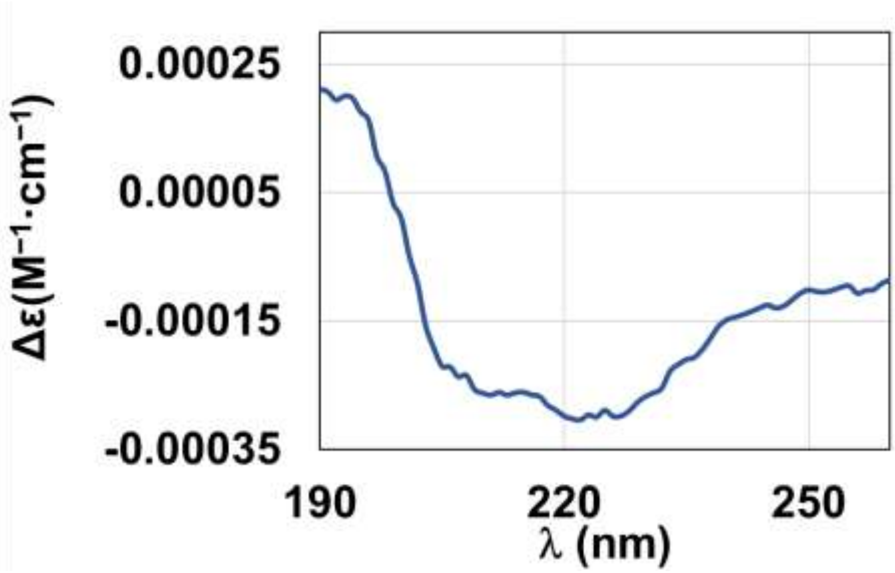

Figure S8. CD curve of stapled peptide Compound **5** in MQ water.

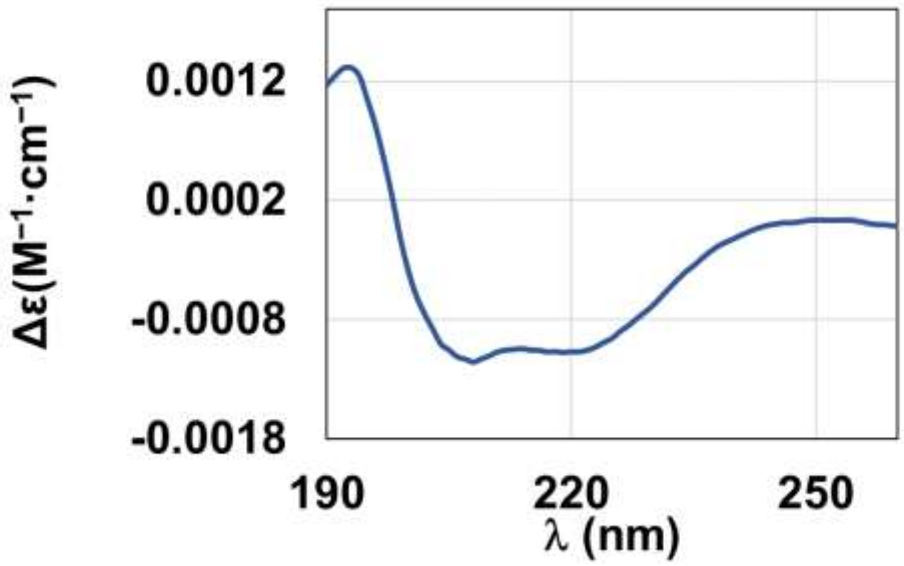

Figure S9. CD curve of stapled peptide Compound **6** in MQ water.

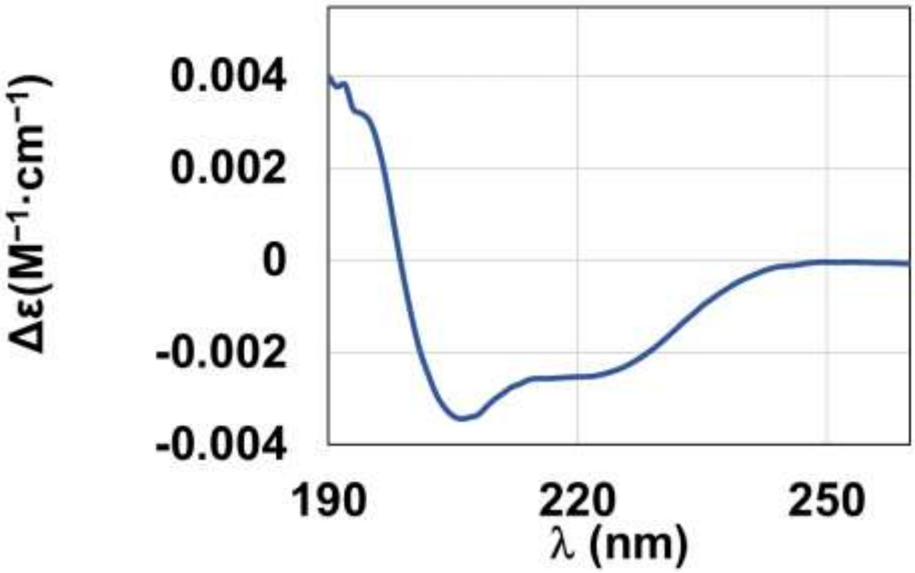

Figure S10. CD curve of stapled peptide Compound **7** in MQ water.

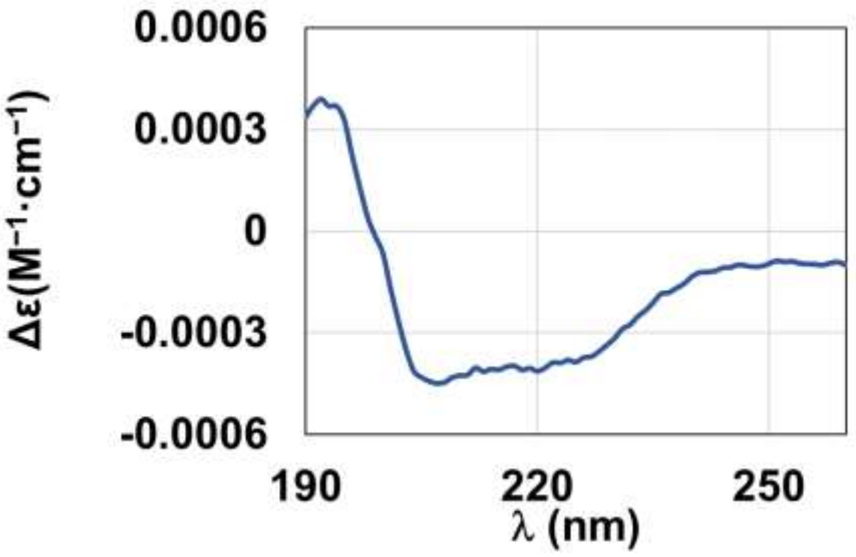

Figure S11. CD curve of stapled peptide Compound **8** in MQ water.

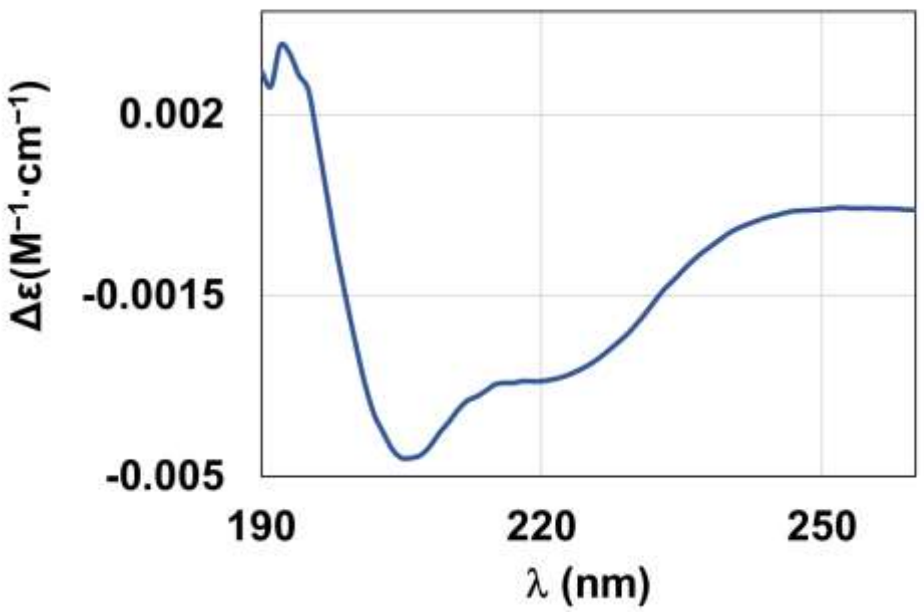

Figure S12. CD curve of stapled peptide Compound **9** in MQ water.

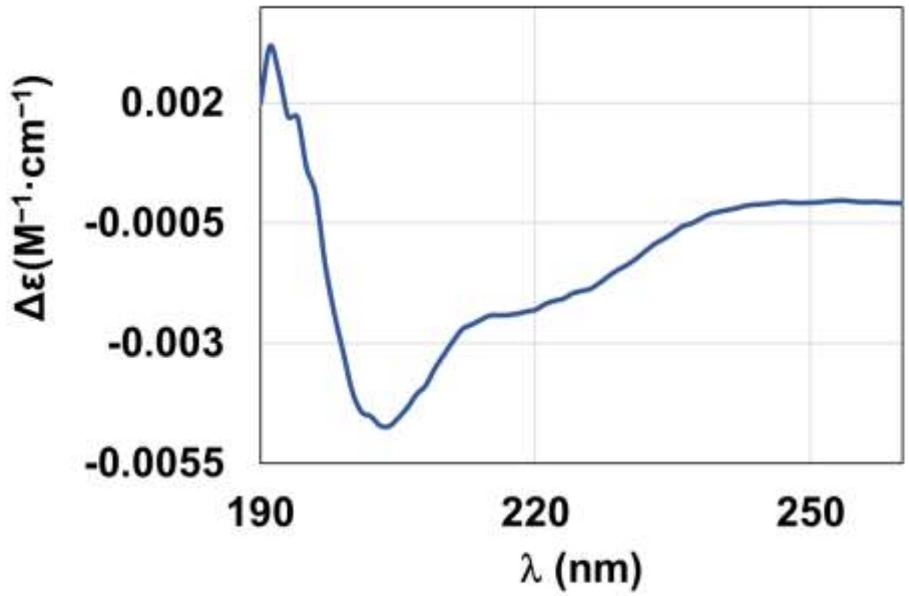

Figure S13. CD curve of stapled peptide Compound **10** in MQ water.

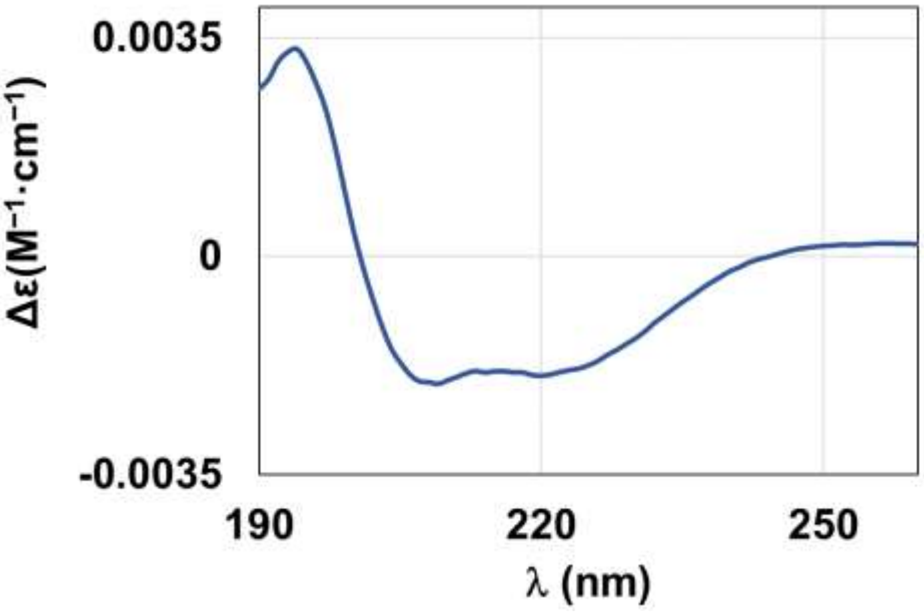

Figure S14. CD curve of stapled peptide **155H1** (buffer: 15mM Phosphate pH 7.5).

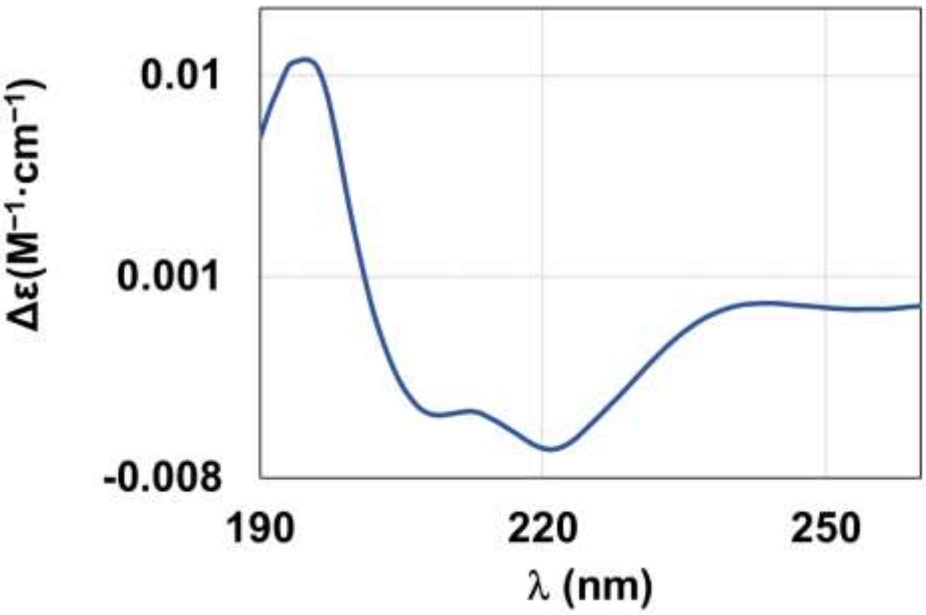

Figure S15. Dose−response DELFIA displacement assay curves comparing the ability of non-covalent analog of stapled peptide **155H1**, to displace the binding of a biotinylated-BH3 peptide to hMcl-1(172-323) (green) or hBfl-1(1-149) (blue). The structure of the non-covalent analog of stapled peptide **155H1** is also reported.

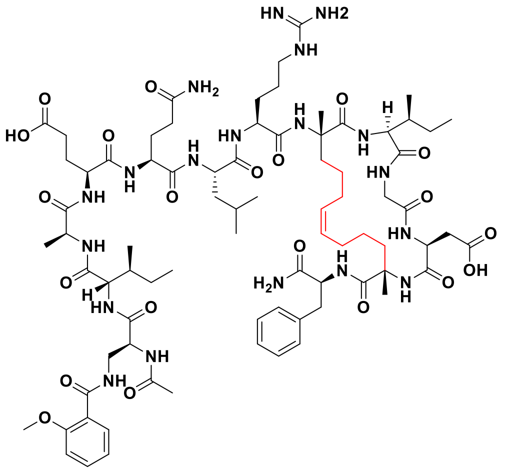

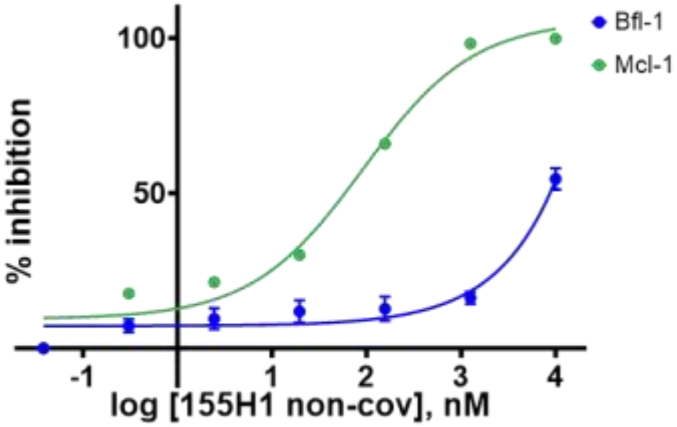

Figure S16. Time-course for the reaction between 155H1 and Mcl-1(172-323) using SDS-Gel electrophoresis and MS analyses. A) SDS gel electrophoresis of wt HisTag hMcl-1(172-323) collected in the absence or presence of stapled compound **11** (**155H1**). The samples for the gels were obtained by incubating 10 μM wt HisTag hMcl-1(172-323) with 100 μM of compound **11** (**155H1**) at room temperature for the indicated incubation time in buffer (50 mM Phosphate pH 7.5, 150 mM NaCl, 1 mM DTT). The two gels were run with the same samples. The percentage of complex formation in time is also reported. B) LC-MS analysis performed to confirm the formation of the covalent complex. 10 μM of hMcl-1(172-323) was incubated with 100 μM of **155H1** at room temperature for the indicated incubation time in buffer (50 mM Phosphate pH 7.5, 150 mM NaCl, 1 mM DTT). The mass of hMcl-1(172-323) is 19,572 Da and 19,750 Da after phosphogluconoylation. The mass of the covalent adduct is 21,307 Da and 21,485 Da after phosphogluconoylation.

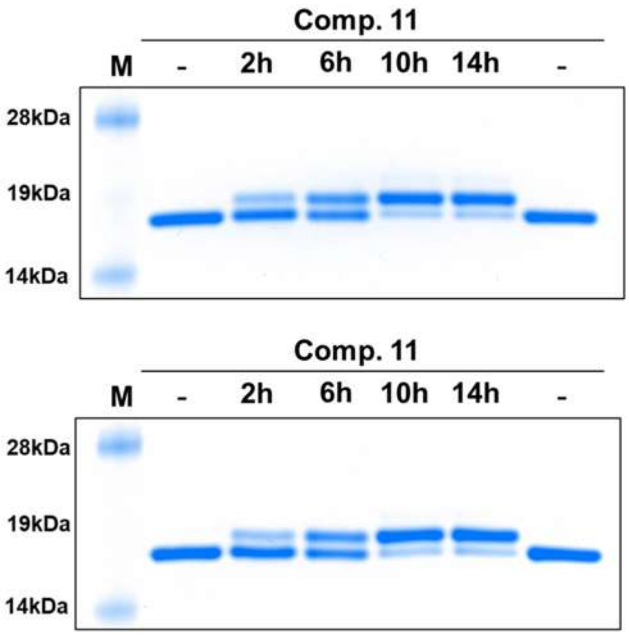

2h:

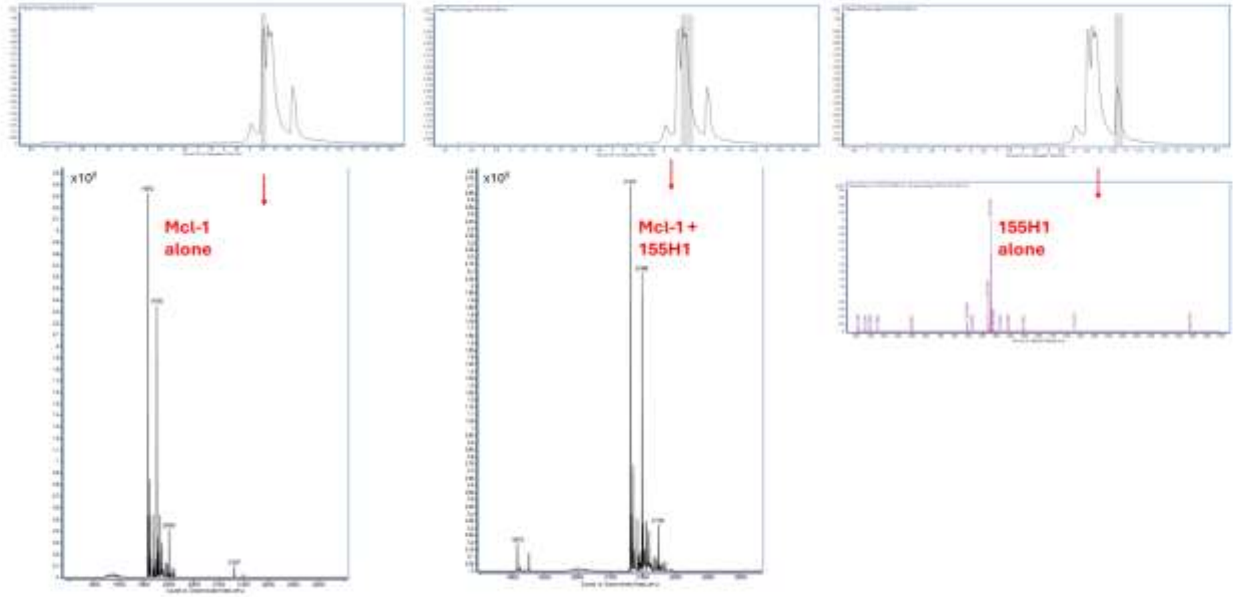

Mcl-1
alone
Mcl-1 +
155H1
155H1
alone

6h:

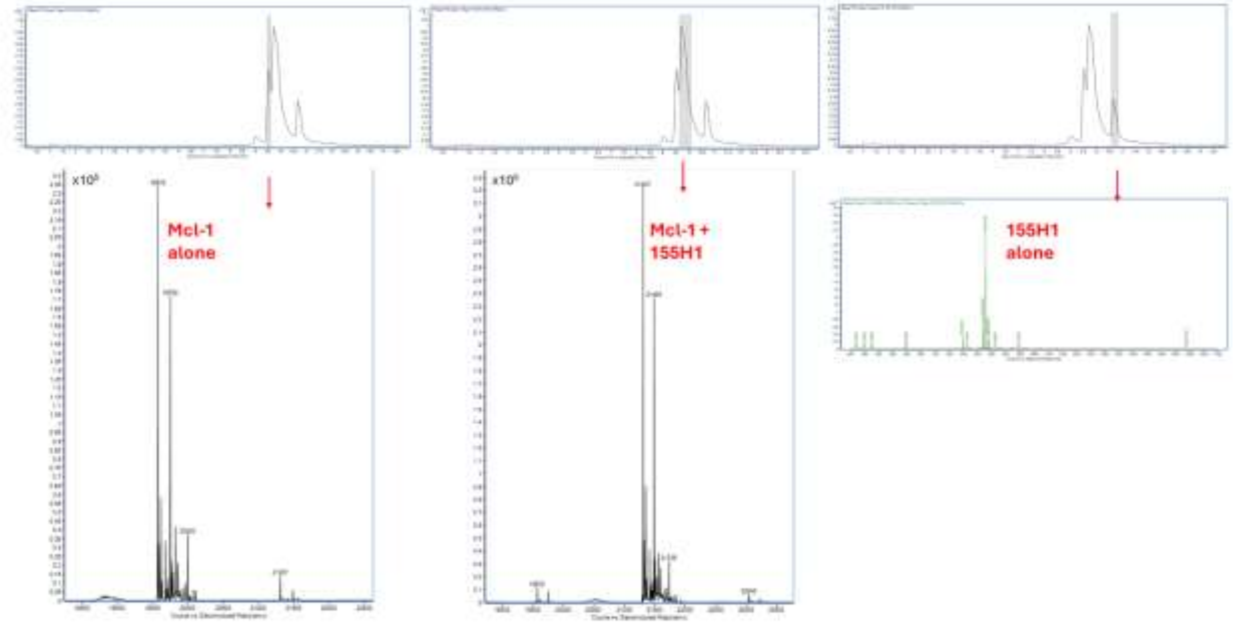

Mcl-1
alone
Mcl-1 +
155H1
155H1
alone

10h:

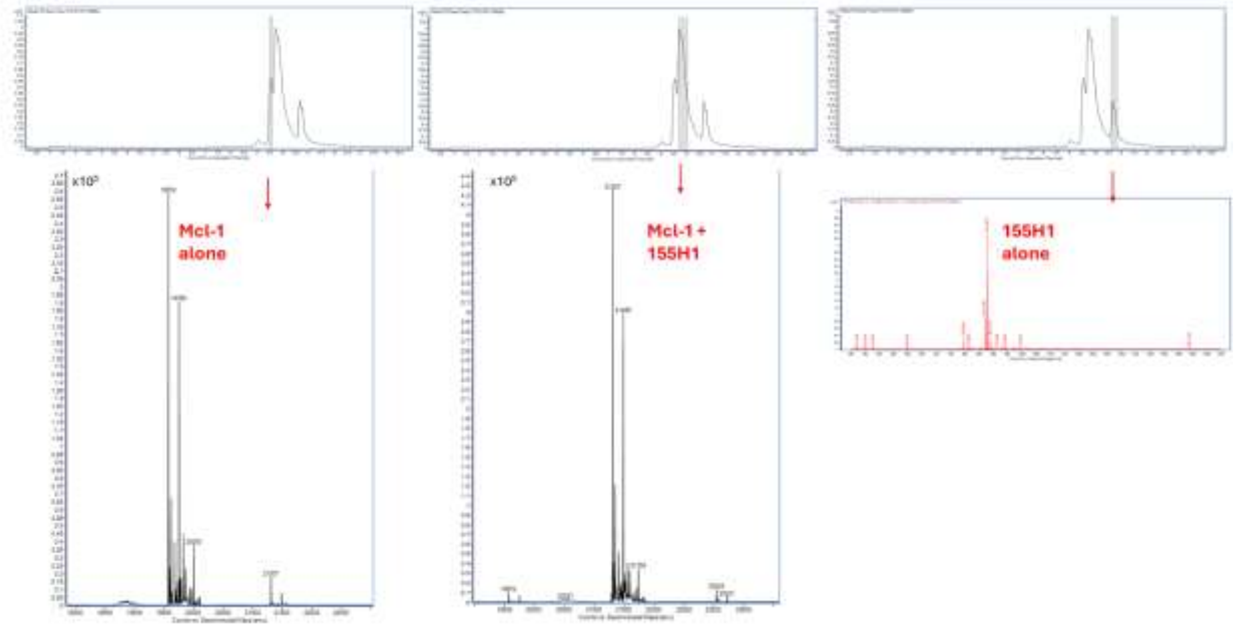

Mcl-1
alone
Mcl-1 +
155H1
155H1
alone

14h:

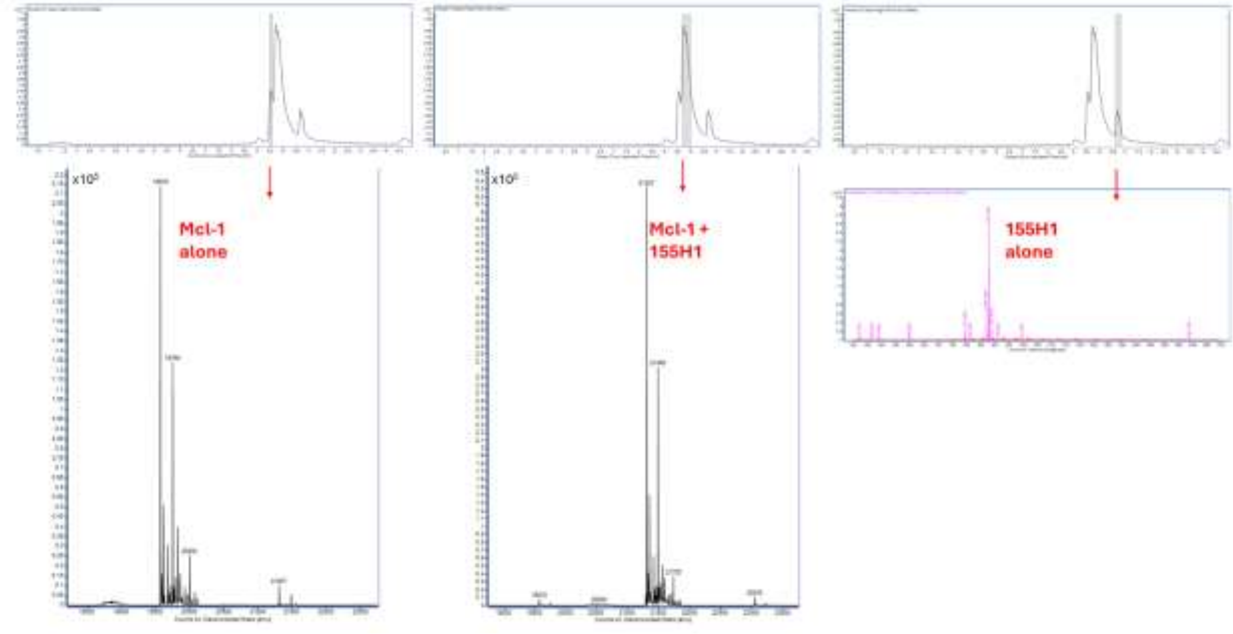

Mcl-1
alone
Mcl-1 +
155H1
155H1
alone

Figure S17. Mass analysis of *wt* hMcl-1(172-323).

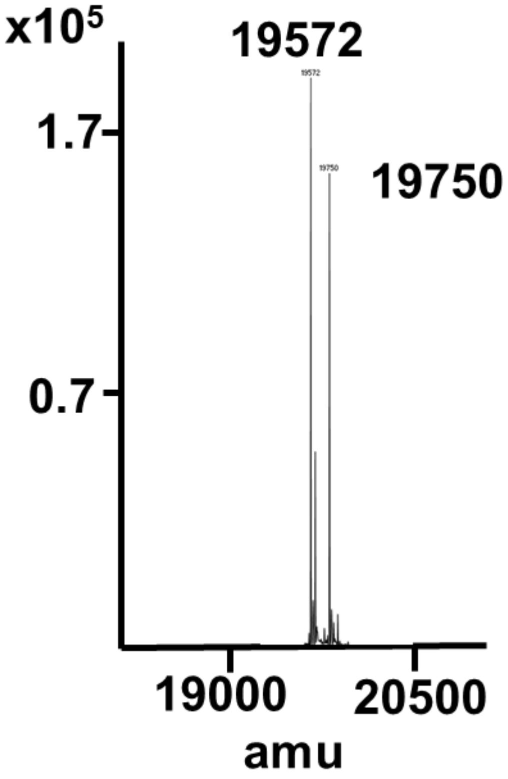

Figure S18. Mass analysis of hMcl-1(172-323) K234A.

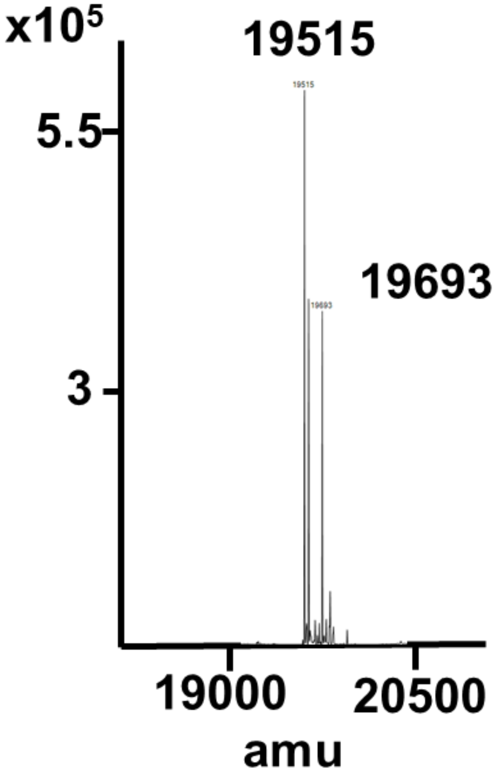

Figure S19. Detection of ligand binding via time-dependent ^{1}H and ^{13}C$^{\varepsilon}$-Met NMR measurement. A) Ribbon representation of *wt* hMcl-1 (beige) and the BIM BH3 peptide (pink) in the binding pocket. Met250 is also highlighted. (PDB ID 2PQK). B) 2D [^{1}H,^{13}C] correlation spectrum of 20 μM ^{13}C^{e}-Met hMcl-1(172-323) (blue) in the presence of 100 μM compound **11** at different time points, as indicated C) Aliphatic region of the 1D ^{1}H NMR spectra of *wt* hMcl-1(172-323) (20 μM) recorded in the absence (blue) and presence of 100 μM stapled compound **11** at different time points, as indicated. The resonance assignment for non-overlapping peaks is also reported.

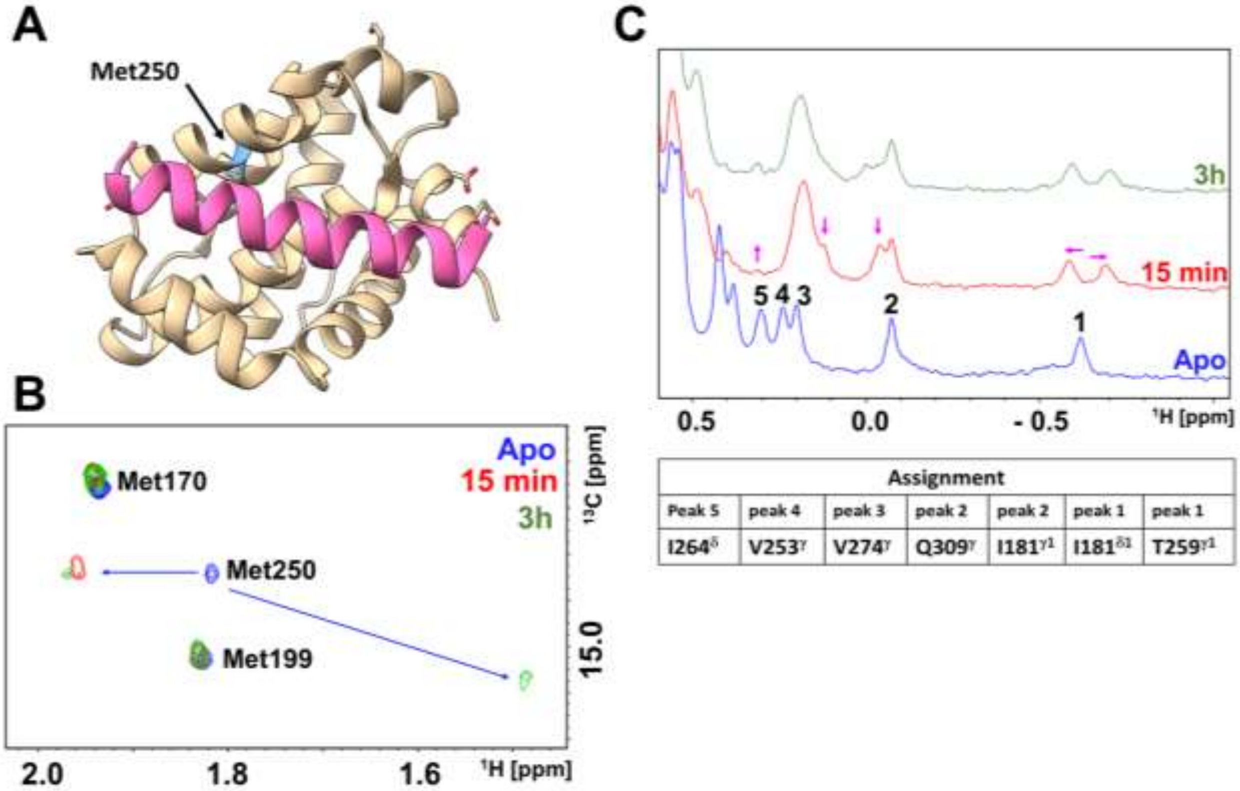

Figure S20. 2D [^{1}H,^{13}C] correlation spectrum of 20 μM $^{13}C^{\varepsilon}$-Met hMcl-1. In the presence of the peptides, Met231's peak broadens behind detection.

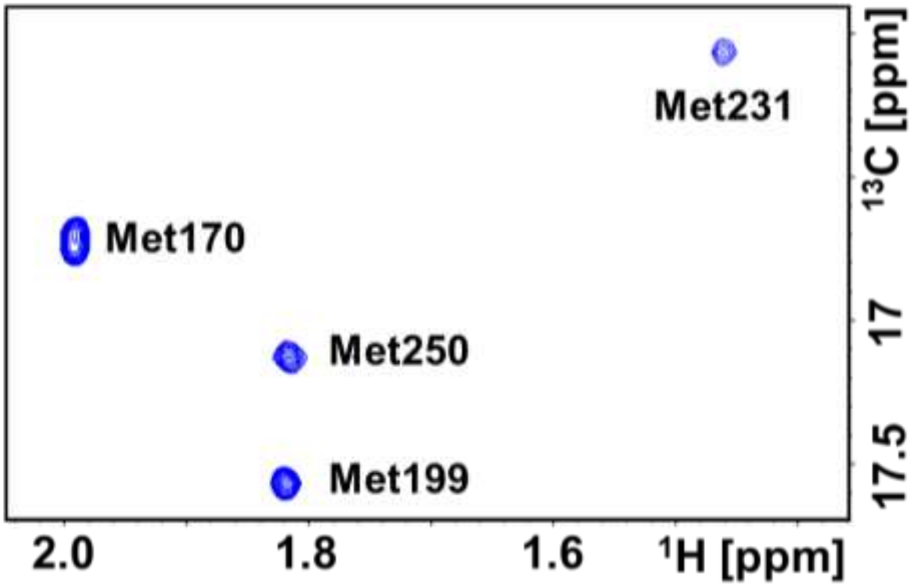

Figure S21. His side chain long range so-fast HMQC [^{15}N,^{1}H] correlations spectra of 50µM *wt* hMcl-1(172-323) (blue) and of 50µM *wt* hMcl-1(172-323) H252A (red). The three cross-peaks characterizing His252 are evident.

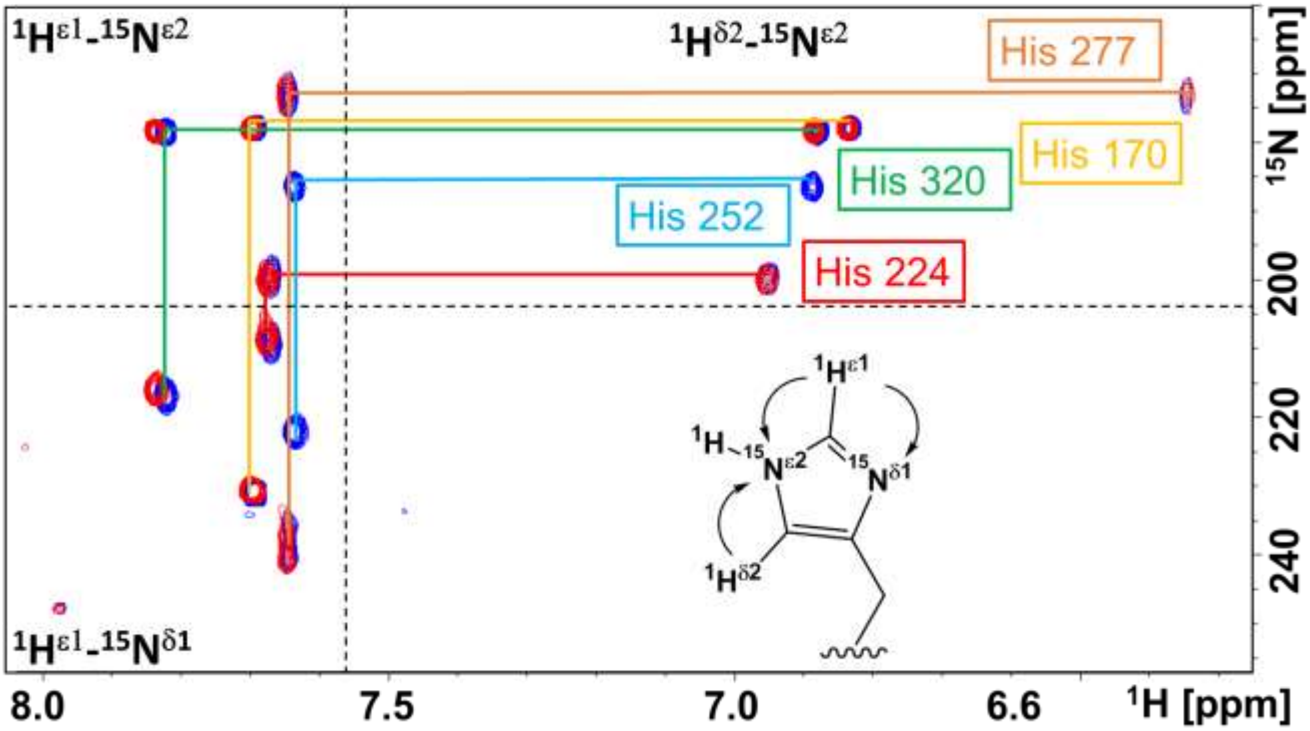

Figure S22. His side chain long range so-fast HMQC [^{15}N,^{1}H] correlations spectra of 50µM *wt* hMcl-1(172-323) (blue) and of 50µM *wt* hMcl-1(172-323) H224A (red). The three cross-peaks characterizing His224 are evident.

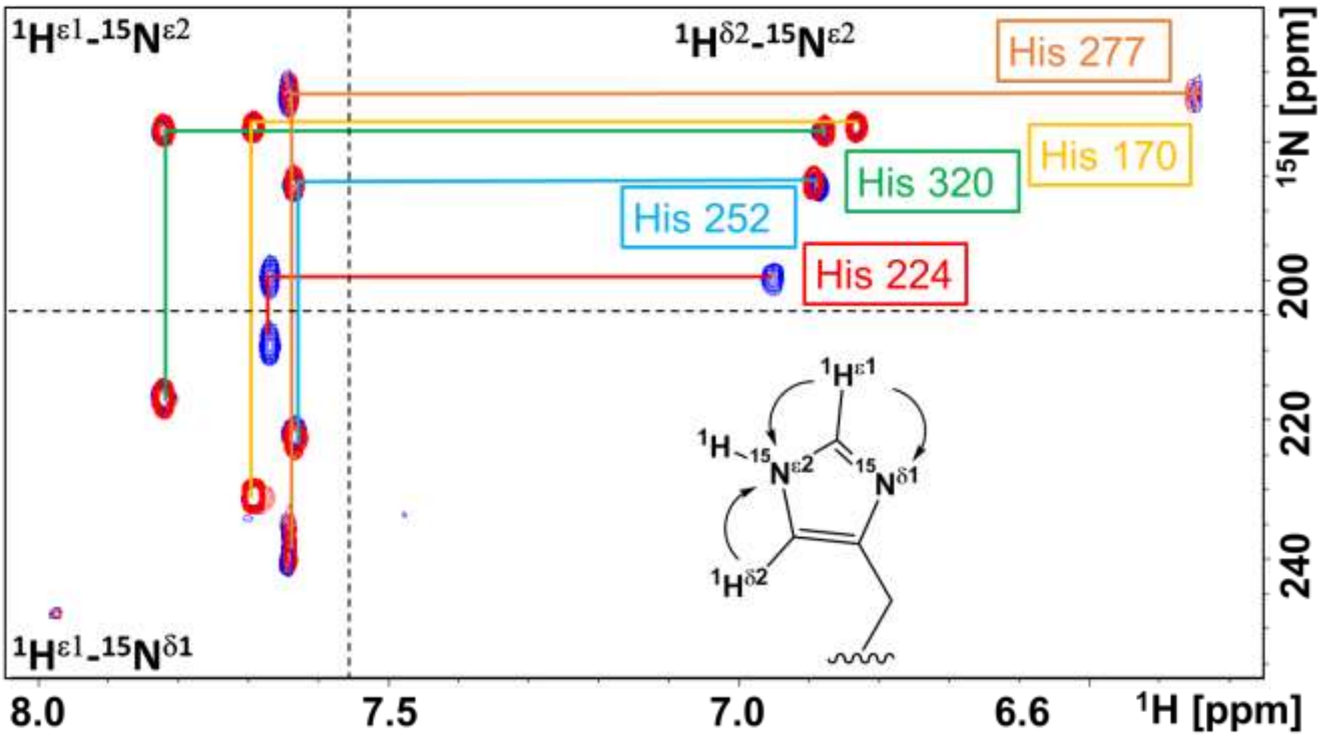

Figure S23. His side chain long range so-fast HMQC [^{15}N,^{1}H] correlations spectra of 50µM *wt* hMcl-1(172-323) H252A in absence (blue) and in presence (red) of 250µM BIM BH3 peptide.

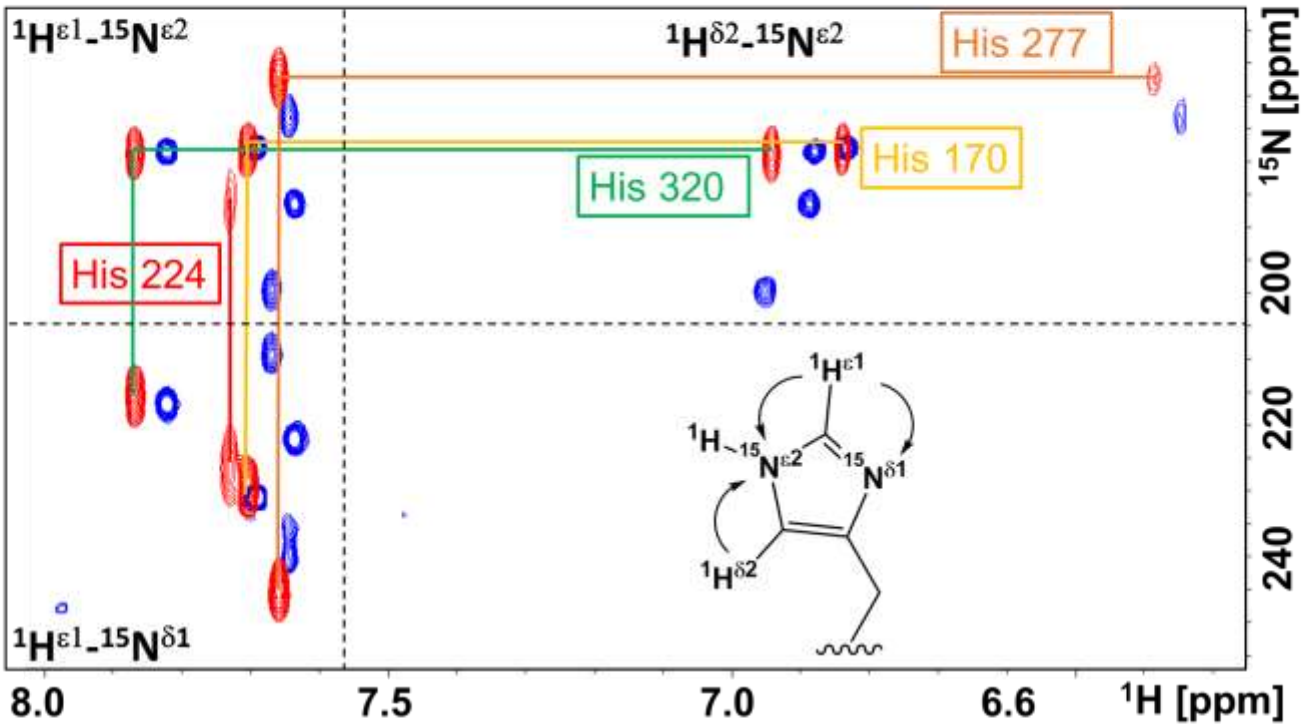

Figure S24. His side chain long range so-fast HMQC [^{15}N,^{1}H] correlations spectra of 50µM *wt* hMcl-1(172-323) H252A in absence (blue) and in presence (red) of 250µM **155H1** stapled peptide.

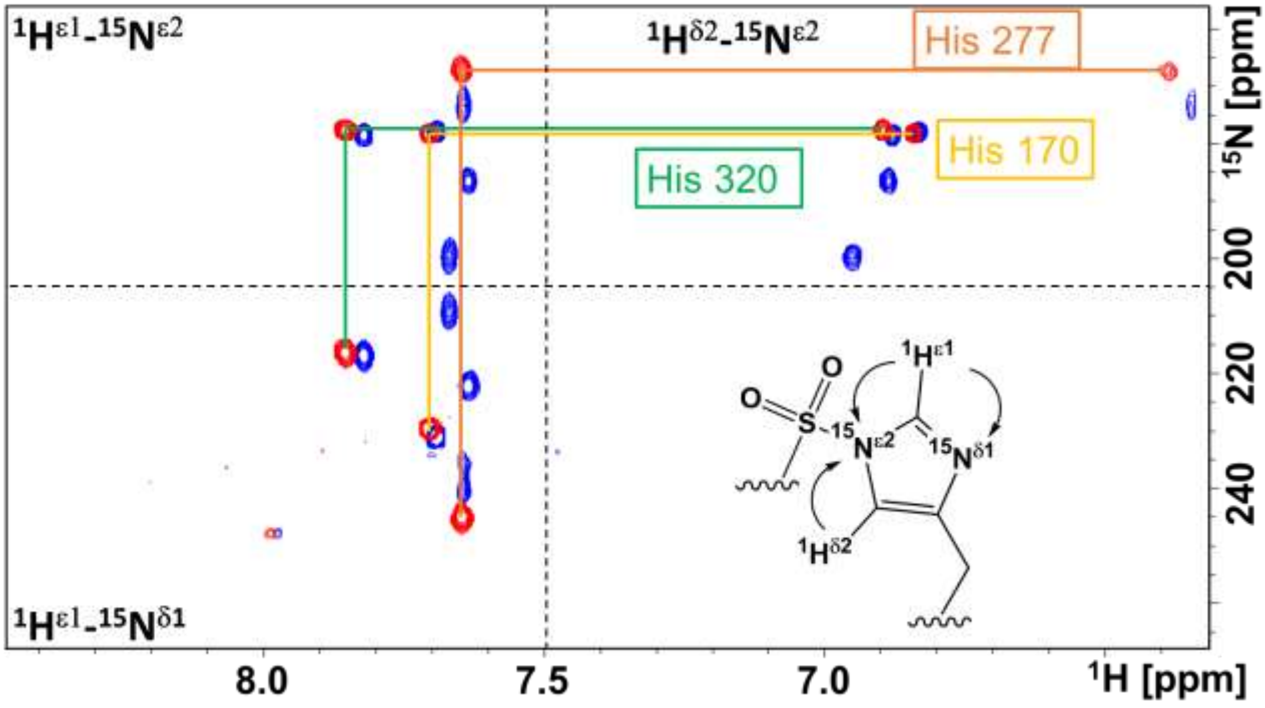

Figure S25. Superposition of the X-ray structures of hMcl-1(172-323) covalently bound to **138E12** (light blue structure. PDB ID 6VBX) and to compound **155H1** (light brown structure. PDB ID 8VJP).

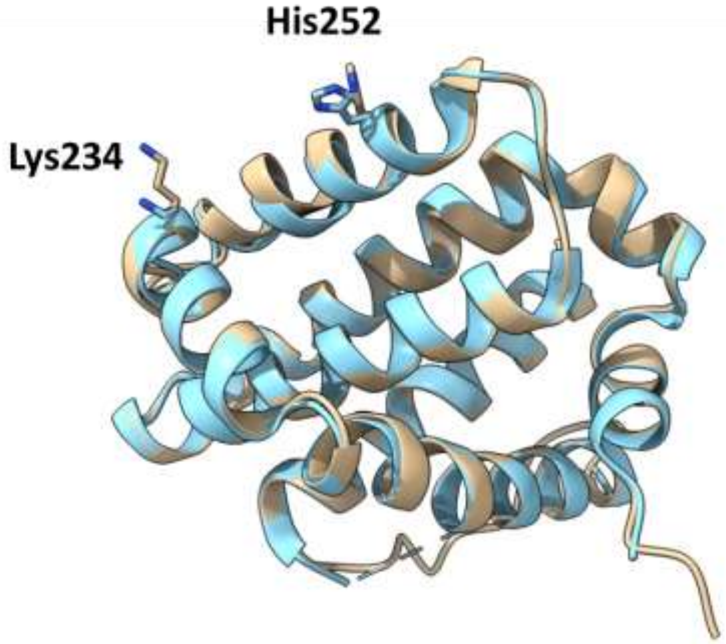

Figure S26. Superposition of the X-ray structures of hMcl-1(172-323) covalently bound to **138E12**, represented in pink (PDB ID 6VBX) and to compound **155H1**, represented in purple (PDB ID 8VJP). A close-up view of the covalent sulfonamide bond resulting from the reaction of the sulfonyl fluoride of **138E12** and **155H1** and the side chain of Lys234 and His252, respectively, are also shown (Panel B).

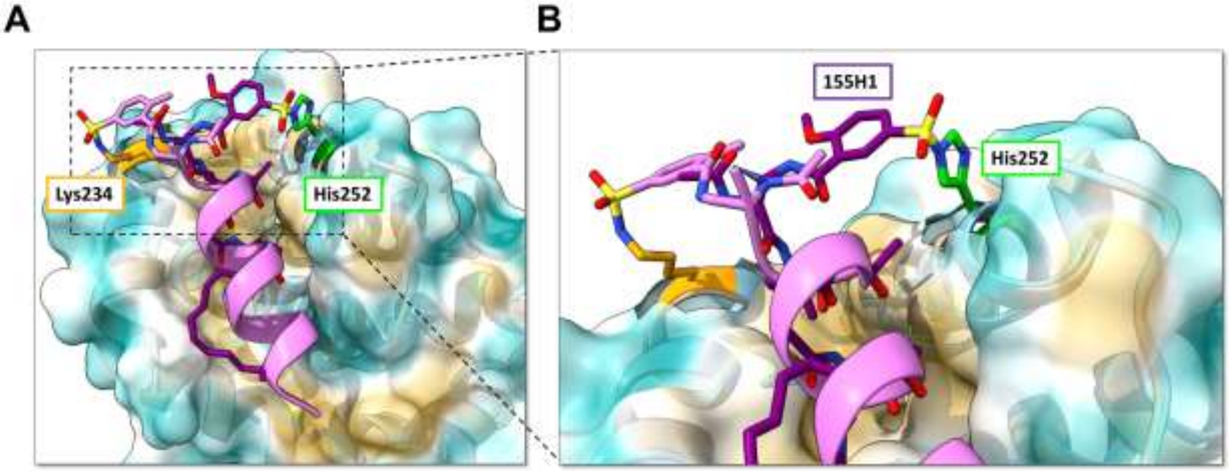

Figure S27. HPLC trace for **155H1** (purity > 98 %).

155H1 analytic 155H1 analytic 1/16/2024 12:31:24 PM

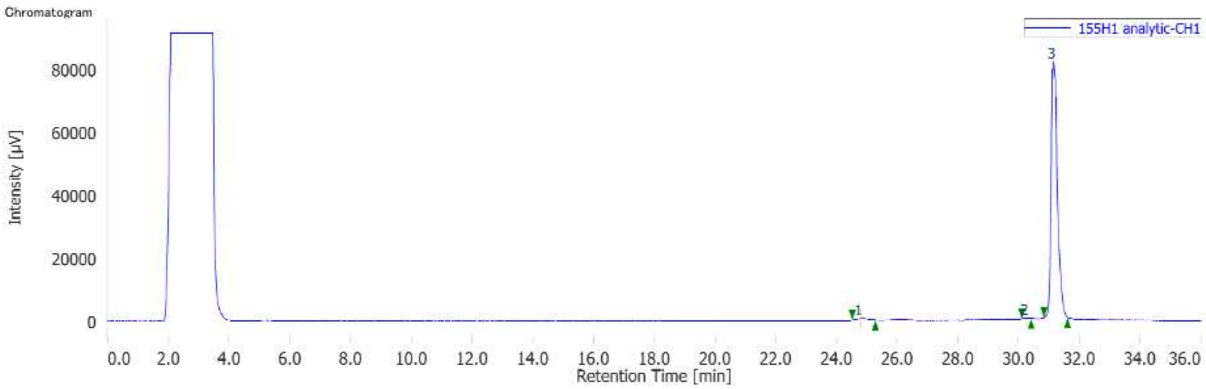

Peak Information

#	Peak Name	CH	tR [min]	Area [μV·sec]	Height [μV]	Area%	Height%	Quantity	NTP	Resolution	Symmetry Factor	Warning
1	Unknown	1	24.788	11938	537	1.015	0.656	N/A	22588	10.155	1.426	
2	Unknown	1	30.247	2234	232	0.190	0.283	N/A	83743	2.380	1.026	
3	Unknown	1	31.145	1162169	81198	98.795	99.062	N/A	135309	N/A	1.452	

Figure S28. Uncropped western blot images.

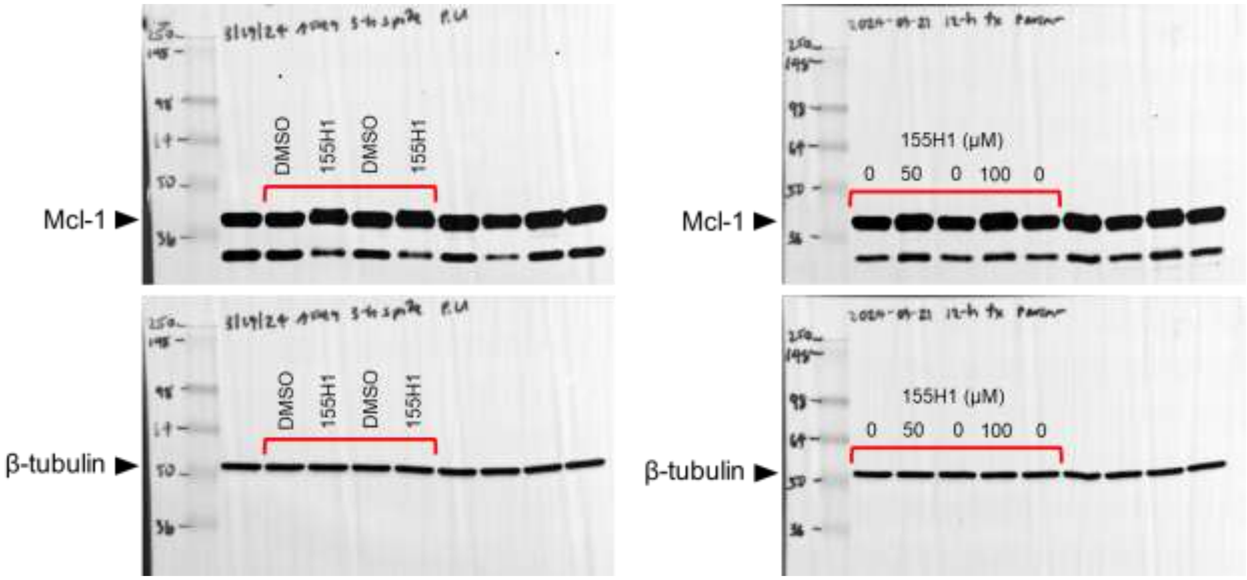

Supplement: Supplementary file 1 — jm4c00277_si_001.pdf [file jm4c00277_si_001.pdf]
